# Super-enhancers complexes zoom in transcription in cancer

**DOI:** 10.1186/s13046-023-02763-5

**Published:** 2023-07-28

**Authors:** MengTing Wang, QingYang Chen, ShuJie Wang, Han Xie, Jun Liu, RuiXiang Huang, YuFei Xiang, YanYi Jiang, DaSheng Tian, ErBao Bian

**Affiliations:** 1 Department of Orthopaedics, The Second Affiliated Hospital of Anhui Medical University, 678 Fu Rong Road, Hefei, 230601 Anhui Province China; 2 Institute of Orthopaedics, Research Center for Translational Medicine, The Second Hospital of Anhui Medical University, Anhui Medical University, Hefei, 230601 China; 3 School of Pharmacy, Anhui Medical University, Hefei, 230032 China; 4 Department of Clinical MedicineThe Second School of Clinical Medical, Anhui Medical University, Hefei, China; 5 Anhui Province Key Laboratory of Medical Physics and Technology, Institute of Health and Medical Technology, Hefei Institutes of Physical Science, Chinese Academy of Sciences, Hefei, 230031 China; 6 Hefei Cancer Hospital, Chinese Academy of Sciences, Hefei, 230031 China

**Keywords:** Super-enhancer, Transcription factors, Histone-modifying enzymes, Cofactors, Noncoding RNA

## Abstract

Super-enhancers (SEs) consist of multiple typical enhancers enriched at high density with transcription factors, histone-modifying enzymes and cofactors. Oncogenic SEs promote tumorigenesis and malignancy by altering protein-coding gene expression and noncoding regulatory element function. Therefore, they play central roles in the treatment of cancer. Here, we review the structural characteristics, organization, identification, and functions of SEs and the underlying molecular mechanism by which SEs drive oncogenic transcription in tumor cells. We then summarize abnormal SE complexes, SE-driven coding genes, and noncoding RNAs involved in tumor development. In summary, we believe that SEs show great potential as biomarkers and therapeutic targets.

## Introduction

Our knowledge of the regulatory genome has recently changed substantially. Enhancers are key cis-acting elements that regulate the spatiotemporal expression of genes [1]. Enhancers are linear distal noncoding regions that stimulate transcription via long-range cis-chromatin interactions independent of their genomic orientation. In addition to typical enhancers (TEs), the genome harbors many linear TEs that often span a few thousand bases known as SEs [2]. SEs constitute a class of cis-regulatory elements with hypertranscriptional activating potential that were first described by Richard A. Young in 2013 [3].

In contrast to those of TEs, the span of SE regions is usually between 8 and 20 kb, much longer than the span of 200 to 500 bp for TEs [4]. The same factors bind enhancers and SEs, including transcription factors (TFs); histone-modifying enzymes (HMEs); cofactors, such as bromodomain and extraterminal domain (BET) family proteins; mediator complexes, cyclin-dependent kinases (CDKs); and RNA polymerases. However, SEs usually recruit more TFs, HMEs, mediator complexes, and RNA polymerases than the other aforementioned factors to regulate the expression of target genes, which can result in powerful regulatory actions [5]. Furthermore, SEs regulate the expression of cell recognition genes, which can be utilized to distinguish cell-type-specific TFs and tumor-specific oncogenes [6]. As research continues to be more intensive, SEs may provide new ideas for developing therapeutic strategies for tumors.

This review provides details and summarizes current knowledge regarding the characteristics and functions of SEs in cancer, including how SEs are generated and identified. Furthermore, this review discusses the deposition and function of the SE complexes mediating protein-coding gene expression and noncoding RNA (ncRNA) regulatory effects in cancer. At the same time, the diverse mechanisms of SE actions in cancer cells are outlined, and the potential use of SEs as biomarkers and therapeutic agents or targets in the clinic is discussed. Finally, this review discusses the major challenges that need to be resolved and knowledge gaps in the biology that need to be filled in this research field and suggests SEs frontiers in clinical practice.

## Characteristics of super-enhancers

**Fig. 1:**
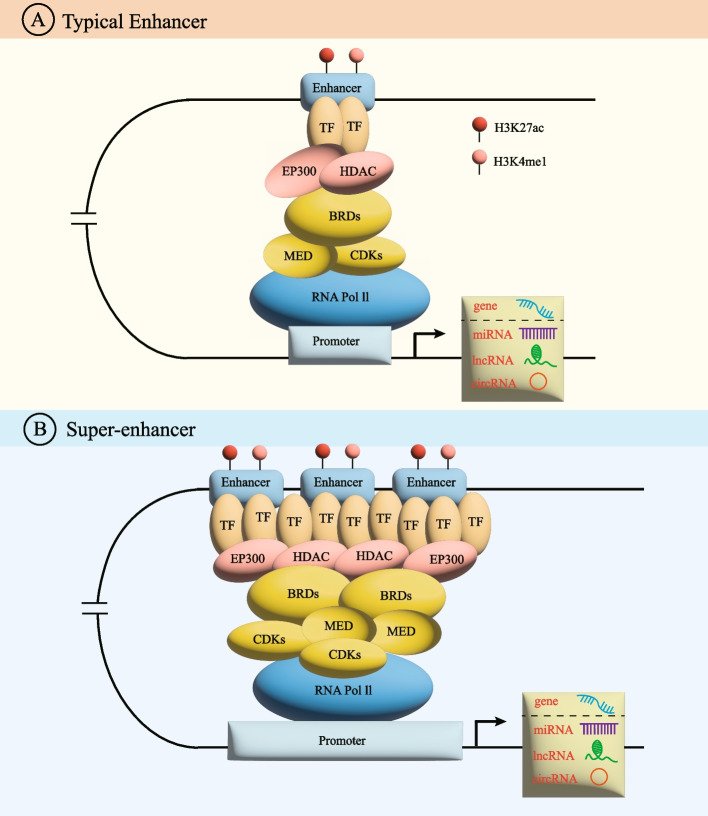
Comparison of typical enhancers and SEs. SEs are enriched with more TFs, HMEs(EP300 and HDAC), and SE-cofactors (BET family proteins, mediator complexes, and CDKs), than typical enhancers. Thus, SEs have stronger regulatory functions than typical enhancers. Furthermore, SE regions bind SE complexes in an order. First, histone acetylating transferases such as p300/CBP are recruited by TFs, promoting nonhistone protein and nucleosome acetylation. Second, BET proteins interact with hyperacetylated histone sites across chromatin, establishing transcriptionally active regulatory regions because of their increased affinity for proteins with many acetylated residues. The establishment of synergistic high-density and cooperative transcriptional complexes at SEs is facilitated by BRD4 and mediator complexes, thereby altering the chromatin structure, dynamics, and function. Finally, a P-TEFb complex and CDK7 work in concert to release RNA Pol II and activate transcription. (BRDs = Bromine domain proteins, CDKs = Cyclin-dependent kinases, HDAC = Histone deacetylase, HMEs = Histone modifying enzymes, H3K27ac = Histone H3 lysine 27 acetylation; H3K4me1 = Histone H3 lysine 4 mono-methylation; P-TEFb = positive transcription elongation factor-b; TF = Transcription factor.)

With cell-specific characteristics and physiologically essential cis-regulatory elements in multicelled creatures, enhancers control gene expression by interacting with adjacent promoters. As specialized enhancers, SEs forcefully boost the transcription of their target genes because they are assemblies of high-density critical TFs and cofactors. An increasing number of studies have revealed that SEs differ from TEs in several ways (Fig. 1). SEs have the following characteristics: (1) the degree of SE mediation is at least one order of magnitude greater than that of TEs; (2) the number of TFs bound by SEs and chromosomes related to transcriptional activity is much higher than that bound by TEs(2); (3) genes regulated by SEs have much higher expression levels than genes regulated by TEs [7]; (4) the SEs that bind to tissue-specific TFs differ, allowing cancer cells to retain their identity; (5) SEs are more likely to disrupt the expression transcription-regulating genes; and (6) SE activity leads to the creation of more enhancer RNAs (eRNAs) than are generated by TEs, these eRNAs show a remarkable ability to stimulate the transcription of many target genes and thus promote gene expression [8].

## The organization, identification and functionality of super-enhancers

As sequencing and analysis techniques have been improved, an increasing number of methods have been invented to distinguish SEs. The invention of ChIP-chip, which is based on a DNA microarray chip, was first used to analyze DNA fragments enriched via chromatin immunoprecipitation (ChIP) in 2000, and the revelation of a transcriptional regulatory code in 2004 laid the foundation for SE identification. In previous studies, chromatin immunoprecipitation sequencing (ChIP-seq), a binding site assay used for studying protein‒DNA interactions in vivo, was used to analyze the abundance of transcriptional activity of molecular markers of enhancers across the genome and to identify active enhancer sites. Then, the activity of the identified enhancers were analyzed, and the SEs were identified. Next, histone modifications were studied. In 2007, transcription elongation was found to be associated with the modification histone H3 lysine 79 dimethylation (H3K79me2), which is associated with transcriptional activation [9]. Nucleosomes located on either side of a TF binding site show unique chromatin-related features, including but not limited to the histone H3 lysine 27 acetylation (H3K27ac) mark, which is a histone modification associated with transcriptional activation, and the histone H3 lysine 4 mono-methylation (H3K4me1) mark, which is a histone modification that is also associated with transcriptional activation. Furthermore, in 2010, the mediator and cohesion complexes were shown to connect gene expression properties to chromosome structure, thereby playing an important role in the discovery and characterization of SEs [10]. In 2013, it was shown that SEs, including those comprising large quantities of critical embryonic stem cell (ESC) TFs, such as Sox2, Oct4, Esrrb, Klf4, and Nanog, drove more-robust transcriptional activity than conventional enhancers and that they are highly susceptible to mediator complex, even at low levels [2]. Subsequently, more SEs were discovered in various cell types due to the concentration of master TFs occupation of cell-type specific genes that govern the biological activity of cells. Through sequencing and bioinformatics analysis, H3K27ac was found to be a better SE marker than other markers such as H3K4me1, DNase hypersensitivity or p300 [11]. The peak obtained by H3K27ac ChIP-seq was analyzed with ROSE software. The adjacent enhancer elements were characterized by peaks obtained by H3K27ac ChIP-seq and sorted according to the signal of H3K27ac in the ChIP-seq data, with high signal strength indicating a SE [12]. However, the characterization of SEs still needs to be updated. Chromosome conformation capture techniques such as chromosome conformation capture sequencing, chromosome conformation capture, and chromosome conformation capture carbon copy sequencing enable people to analyze the three-dimensional structure of the nucleus at super resolution and perform high-throughput sequencing. As the number of SE identification methods increases, SE characteristics and complexity will also be increasingly revealed.

After Yin Chen proposed the concept of a SE, Richard A. Young created a new method to identify SEs; at this time, most studies on transcriptional regulatory elements of genes were limited to transcription start sites (TSSs) [3, 13]. These regulatory sequences are generally short and next to a promoter. A luciferase reporter system can be used to construct a whole "enhancer + promoter" sequence directly to enable study on an enhancer function. However, a SE spans a large portion of the genome (approximately K to hundreds of K), often stretching far from the site it regulates, making it unreasonable to adopt the traditional "enhancer + promoter" model. First, a SE span is larger than the span of a plasmid; second, the model would artificially place the SE close to a promoter (not next to the genome but artificially assembled on the genome), which does not eliminate the possibility that a separate SE could regulate the distal promoter. Therefore, we needed to master how to verify SE function after obtaining ChIP data. (1) When a SE span is large and a SE comprises a TF regulatory network, to confirm that the SE regulates distal genes, the expression level of targeted genes would be measured with and without the SE. Using H3K27ac ChIP-seq data, Li et al. identified regions located downstream of the Sox2 locus where a SE potentially functioned [14]. A cas9 targeting each side of a SE was designed, CRISPR removed the SE; the absence of the SE was verified by the reduced expression of Sox2 and several pluripotency factors was reduced. (2) When a SE span is short, core sites or clear, and because abnormal SE function is caused by factors such as mutation, SE function can be investigated by reconstructing the local epigenetic marks [15]. By rebuilding the epigenetic landscape of a target genomic locus, Li et al. revealed that the combination of separate repressors (KRAB and LSD1 in an enhancer-targeting CRISPR epigenetic editing repression system for efficiently analysis of enhancer function in situ and in vivo) or activators (VP64 and p300 in an enhancer-targeting CRISPR epigenetic editing activation system (enCRISPRa) to analyze enhancer function in situ and in vivo) led to more effective gene transcription regulation. For example, in xenografts, the enCRISPRa allele was precisely localized to a carcinogenic TAL1 SE and shown to govern TAL1 expression and tumor growth [16].

SEs are cell-type-specific TE clusters with biological activities realized by promoting the expression of critical cell-identifying genes [2]. SEs indicate cell identity, sustain cancer cells, and be evaluated to discriminate between cancer subtypes [17]. Diverse SEs facilitate gene regulation through their interactions with specific sites with different activation levels at developmental stages or through synergistic gene expression [18]. Furthermore, cancer-linked somatic mutations typically arise in SE-enriched genomes and are regulated by SEs. Numerous cancer-related studies have described "enhancer hijacking," a process in which SEs as multifaceted regulatory elements activate oncogenes in various biological contexts. For instance, research on adenoid cystic carcinoma revealed that SE translocation promoted the overexpression of oncogenic TFs in cancer cells [19]. In addition, through eRNA synergy or TF redistribution, SEs can govern cellular characteristics by activating cell type-specific signaling pathways and stimulating or inhibiting cell maintenance-related genes in response to stimuli. SEs, which function as dynamic regulatory components, are crucial for controlling the identity of tumor cells and influencing how they react to their environment [20]. In addition to their regulatory functions in many tumor cell types, recent research has revealed that SEs play a role in the genome of tumor cells [17]. However, the processes underlying SE functions in specific tumor cells are unclear, and SE involvement in genome control still needs to be thoroughly clarified [21]. Therefore, research focused on the regulatory mechanisms of SEs will remain important in the future.

## Regulatory functions of super-enhancers in tumors

### Basic regulatory mechanisms of super-enhancer functions in gene transcription

In eukaryotes, enhancer–promoter interactions are critical to gene transcription; the physical contacts between enhancers and promoters convey the regulatory information required for transcription. The regulation of gene transcription mediated by SEs largely depends on the targeted TFs and cofactors and enhancer–promoter interactions [22]. In a SE, which is a large multiple-enhancer cluster, each component enhancer can independently combine with TFs and cofactors and jointly regulate the transcriptional activity of the same promoter. Therefore, the primary regulatory mechanisms underlying a SE function also depend on the function of its component enhancers and, ultimately, on the interactions between enhancers and promoter function [23]. Furthermore, numerous investigations have shown that enhancer–promoter links are formed in association with gene activation; however, it is unclear whether these interactions are results or causes of gene activation [24]. The connection between enhancers and promoters is influenced by topologically associating domains (TADs). TADs were the first chromatin structural units found in mammalian cells and are essential to the eukaryotic genome. Evidence shows that a TAD boundary can function as an insulator to prevent inappropriate enhancer–promoter interactions [25]. In this respect, related studies have proven that structural proteins are at the boundary of TADs and are integral to construct chromatin; moreover, structural proteins are abundant on the edge of enhancers, confirming that the CCCTC binding factor, a protein regulating transcription of many viruses, is at the boundary between TADs and enhancers [26]. A study on an imbalance in the function of breast cancer SEs showed that the crucial TF RUNX3 occupies a distal SE, which regulates the expression of the RCAN1.4 gene, and the low expression of this TF in breast cancer leads to the abnormal action of this SE, resulting in a decrease in RCAN1.4 expression [27]. In addition, the core promoter region and the distal enhancer region of RCAN1.4 were found in the same TAD when additional causes for the low expression of the RCAN1.4 gene in cancer tissues was investigated. This discovery partially reflected the regulatory mechanism involving TADs and enhancers. In summary, the mechanism underlying TAD effects on SEs is different in different situations. However, all the data indicate that SEs may rely on specific TADs to realize some of their transcription activation functions [28].

Previous studies have reported that SEs control oncogenes and that H3K4me3 peaks are greatly enriched at tumor suppressor genes, suggesting that some genomic elements may both promote and inhibit tumorigenesis. This finding also helps explain why SEs may localize to tumor suppressor genes and oncogenes. Several recent studies have shown that SEs are frequently associated with tumor suppressor genes. For instance, the marked overexpression of SE-associated tumor suppressor genes (ETV6, IRF1, IRF8, and CEBPA) inhibited the proliferation of AML cells [29]. In addition, disrupting the expression of the tumor suppressor gene RCAN1.4 promoted the development of breast cancer [27]. *Mll4* deletion downregulated tumor suppressor genes (Bcl6 and Dnmt3a) in medulloblastoma by decreasing SE function [30].

### Mechanism by which super-enhancers drive oncogene transcription

**Fig. 2:**
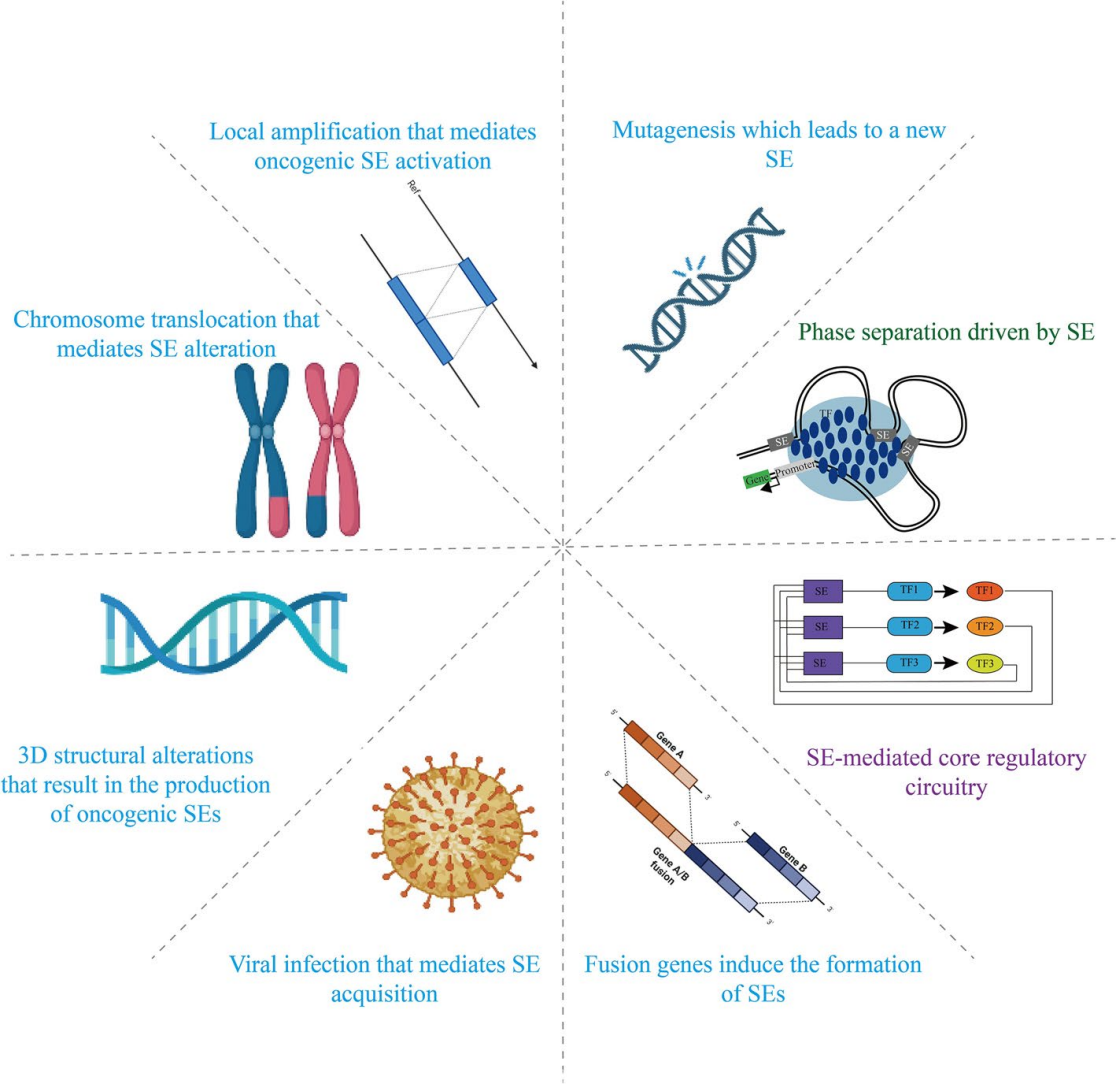
Various potential factors for SE formation: Mutagenesis which leads to a new SE; Local amplification that mediates oncogenic SE activation; Chromosome translocation that mediates SE alteration; 3D structural alterations that result in the production of oncogenic SEs; Viral infection that mediates SE acquisition; Fusion genes induce the formation of SEs; Phase separation driven by SE; SE-mediated core regulatory circuitry. (SE = Super-enhancer)

Genetic research has revealed disease-associated mutations in ncRNA sequences in genomes [31]. Somatic cells can acquire SEs through gene deletion, duplication, translocation, insertion, inversion, and gaining single-nucleotide polymorphisms (SNPs). These gene changes can disrupt the binding of SEs to TFs, thereby affecting the number of SEs replicated and the size of the genome, which can stimulate or inhibit SEs action, disrupting genes adjacent to target genes. Tumor cells acquire these unique SEs during carcinogenesis, and they enhance oncogene expression, activate signaling pathways, and expedite cancer formation [32]. To date, SE-mediated abnormal transcription is thought to be caused mainly by the following factors (Fig. 2): (a) mutagenesis which leads to a new SE; (b) local amplification that mediates oncogenic SE activation; (c) chromosome translocation that mediates SE alteration; (d) 3D structural alterations that result in the production of oncogenic SEs; (e) viral infection that mediates SE acquisition; and (f) fusion genes induce the formation of SEs.

#### Mutagenesis which leads to a new SE

Mutations in a SE DNA sequence may lead to a change in the function of a promoter and the SE. In T-cell acute lymphoblastic leukemia (T-ALL), a 2–18 bp fragment is inserted into the noncoding intergenic region of the TAL1 gene to create a de novo binding site for MYB, thereby initiating SE formation and increasing TAL1 expression [33]. In addition, SNPs have been found to initiate cancer-promoting SE activity. For instance, the acquisition of the LMO1 oncogenic locus in neuroblastoma relies on the conserved binding site of GATA3 combined with GATA. A SNP near a SE converts a preserved GATA-binding site to a TATA motif, substantially reducing LMO1 expression and SE activity [34]. Furthermore, SNPs promote tumor growth by eliminating SEs linked with tumor suppressor genes. Some evidence has linked the BMF gene 15q15.1 risk locus to an increased chronic lymphocytic leukemia (CLL) risk. The SNP at the 15q15.1 risk location can boost the anti-apoptotic activity of BCL2 by mediating the mutation of the proapoptotic gene BMF and the TF RELA recruitment to a SE in CLL [35].

#### Local amplification that mediates oncogenic SE activation

The amplification of copy number mutations, including those encoding SEs, is a common mechanism of oncogene activation. Studying 12 different tumors using tissue-specific epigenetic analysis, Zhang et al. identified SEs near the oncogenes KLF5, USP12, PARD6B, and MYC and determined that they were amplified using somatic copy number analysis [36]. These amplified oncogenic SEs have been seen in various tumor types. For example, near the 3′-end of the MYC locus, SEs are locally amplified at distinct locations in cervical cancer, acute myeloid leukemia (AML), lung adenocarcinoma (LUAD), and acute lymphoblastic leukemia, and thus likely controlling MYC gene expression via germline-specific chromosomal looping [36–38]. Similarly, a 350 ~ 2000 kb genome region carries the MYCN oncogene and its similarly amplified SEs in neuroblastoma, leading to high MYCN expression and driving carcinogenesis [39]. These studies suggest that the local amplification of SEs near genes may be critical for oncogene overexpression.

#### Chromosome translocation that mediates SE alteration

SE may be activated via chromosomal translocation through which it is moved from its normal genome location to an oncogene region. This pattern, dubbed "SE hijacking," has been identified in multiple cancers, including colorectal cancer, AML, medulloblastoma, and neuroblastoma. An established example is the 9 kb inversion in AML cells that transfers a SE from an EV1-activating oncogene to the EV1 gene enhancer, thereby lowering the expression of tumor suppressor factors and activating the oncogene [40]. Furthermore, the MYB gene is moved closer to a distal SE via a chromosomal rearrangement, resulting in increased MYB expression in adenoid cystic cancer (ACC) [41]. Chromosomal translocation in medulloblastoma causes the transfer of the proto-oncogenes GFI1 and GFI1B to sites enriched with activating SEs, which play pro-oncogenic functions. Recent studies have shown that most chromosomal translocations that cause GFI1/GFI1B activation do not include the gene itself, suggesting that this "enhancer hijacking" phenomenon is equally common in other solid tumors [42].

#### 3D structural alterations that result in the production of oncogenic SEs

Chromatin forms a series of hierarchical three-dimensional (3D) domains in the nucleus during interphase; these domains include regions that can auto-interact called TADs. TAD boundary disruption, via genetic or epigenetic changes, allows new genes and SEs to be translocated to areas associated with SE hijacking, altering regulatory linkages and triggering cancerogenesis. In recent years, researchers have identified a TAD boundary deletion event through a pancancer analysis using the Cis Expression Structural Alternation Mapping computational framework [43]. Active chromatin spreads to adjacent fusion genes, producing SE elements that increase IRS4 gene expression in squamous cell carcinoma (SCC) cells [44]. Moreover, further studies on chromosome 11 have shown that tandem repeats of the IGF2 gene allow the expansion of the IGF2 gene and the translocation of SE fragments near the boundaries of the closest TAD, forming new 3D regions between preexisting structures that include SE TADs and leading to the abnormal transcriptional regulation of oncogenes [45]. These results suggest that alterations in the 3D architecture can produce oncogenic SEs and induce the overexpression of intracellular oncogenes to trigger tumorigenesis.

#### Viral infection that mediates SE acquisition

A viral infection can lead to the production of SEs, which may then be involved in an increase in the transcription of genes related to cell survival and reproduction. Epstein-Barr virus (EBV), human hepatitis B virus, human papillomavirus (HPV), and T-cell lymphotropic virus type 1 (HTLV-1) are all cancer-causing viruses. EBV infects human B cells to produce EBNA2, 3A, 3C, and EBNALP. These oncoproteins bind to SEs, trigger the transcription of anti-apoptosis and prosurvival genes such as BCL2, IKZF3, MIR155, and MYC, and promote the proliferation of lymphoblastic cells [46]. Research revealed that EBV SEs are transcribed into eRNAs, which increase the oncogene transcriptional activity of MYC [47]. Previous studies have shown that the high-risk HPV cancer protein E6 activates SEs in cervical cancer cells and promotes the development of tumors by regulating histone demethylase KDM5C expression [48]. Furthermore, HBZ is the only HTLV-1-encoded TF that is expressed in all adult T-cell leukemia/lymphoma (ATLL) cases. HBZ binds to an ATLL-specific BATF3 SE and controls the expression of BATF3 and its downstream targets, including MYC, thereby promoting the proliferation of lymphoma cells [49].

#### Fusion genes induce the formation of SEs

Gene fusion refers to a sequence in which some or all of the sequences of two genes are fused through some mechanism (such as genome variation) to form a new gene [50, 51]. A fusion gene can induce the production of SEs, thereby driving the transcription of oncogenes. Benbarche et al. discovered a novel SE called SE KIT, which was formed by the ETO2-GLIS2 fusion gene, that regulates the expression of adjacent PDGFRA and KIT genes. Further research indicated that this SE is unique to this leukemia subtype, that an ETO2-GLIS2 fusion can activate the SE and that the SE can coregulate two tyrosine kinase genes in a signaling pathway that controls the progression of leukemia [52]. Additionally, hybrid SEs produced by the C19MC-TTYH1 gene fusion increase the C19MC-LIN28A-MYCN carcinogenic circuitry, promoting the original malignant epigenetic state of multilayer rosette embryonic tumors [53]. Moreover, overexpression of the fusion gene TMPRSS2–ERG leads to the formation of a SE, which promotes the transcriptional activity of related genes in prostate cancer (PCa) [54]. These findings suggest that powerful fusion oncogenes may cause cancer by inducing SE formation.

### Super-enhancers mediate transcriptional control through phase separation

Phase separation (PS), also known as phase transition, is a common well-characterized physical phenomenon [55]. Brangwynne et al. discovered that P particles (proteins) smash into one another similar to droplets, forming small scattering droplets, and then swiftly fuse to produce enormous droplets in *Caenorhabditis elegans* Ppins [56]. This study was the first to confirm phase transition-based processes in living organisms. Further studies revealed that PS was involved in other interactions cells, such as those among nucleoli and proteins, and in life-supporting activities, such as DNA repair and neural signaling [57, 58]. PS suggests a new theoretical model for the complex gene regulating functions of eukaryotic DNA regulatory elements, RNA regulatory molecules, and multiple protein regulatory mechanisms that coordinate with each other [59]. Due to the large amounts of RNA Pol ll and other mediators, these intricate DNA regulatory elements are attracted to each other, and PS of in SEs has been observed. High concentrations of RNA Pol ll carboxy-terminal domain (CTD) and mediators form a condensate through multivalent interactions to form liquid droplets. Target genes of SEs are associated with condensates during transcriptional activation, as demonstrated by Cho et al., and cells treated with 1,6-hexadiol or mediator subunit BRD4 inhibitors prevented the formation of Pol II aggregates and mediator complexes [60]. The quantities of Pol II and mediators in SE condensates were decreased via PS/condensation inhibition, and the target gene transcription rate was also decreased [61]. Additionally, interactions of high-density transcriptional regulators at SE sites led to the PS of multimolecular complexes and to frequent transcriptional bursts [19].

PS promotes aberrant transcription of target genes through SEs implicated in tumor growth. A recent study revealed that the transcriptional coactivators MED1 and BRD4 were separated from SEs via PS and then formed droplets, ultimately separating transcription-related components from the nucleus and realizing the regional compartmentalization of transcription [62]. Another study found that the DNA-binding domains of the EWS-FLI1 fusion protein localized to specific SE regions, and phospholipase D induced PS and activated the expression of the EWS-FLI1 fusion target gene in Ewing sarcoma [63]. In contrast, phase-separation abnormalities may inhibit the development of certain cancers. In multiple myeloma (MM), BRD4 and mediators occupy large regions covered by SEs and drive the expression of signature genes, such as the Myc oncogene [64]. Recent studies have focused on inducing the PS of regulatory tumor oncogenic factors driven by SEs. However, the PS of regulatory tumor suppressors via SEs has been rarely reported.

### Super-enhancer-mediated core regulatory circuitry in tumors

A core regulatory circuit (CRC), known as the SE–TE regulatory network, is chiefly composed of a core TF and a SE regulatory loop. It is widely believed that SEs can regulate not only core TF gene expression alone but also core TF genes together with other TF genes [65]. Notably, many studies have revealed remarkable features of a CRC: (a) each CRC TF is automatically regulated when bound to its SE; (b) a CRC TF binds with the SE of another core TF to form an integrated autoregulatory loop; and (c) the CRC TFs act upon their target genes. According to these characteristics, two mathematical methods, "CRC Mapper" and "Cortosaurus," have been designed to identify CRC TFs and networks. The most significant differences between the two methods are that SE are partitioned via sequential motif analysis or via the automatic adjustment of master TFs [66]. In contrast to the complete SE domain identified by "CRC Mapper," "Coltron" scans the nucleosome-free region (NFR) [67]. Additionally, "Coltron" needs at least one TF-binding motif in the NFR of the SE, and "CRC Mapper" requires three TF-binding motifs [68]. Hence, to use "Coltron", additional chromatin accessibility data, such those obtained by ATAC-seq or DHS, are needed to annotate the NFRs. These approaches are clearly essential since they have been used to identify a range of certain CRCs. A CRC is involved in the development of several types of tumors, including neuroblastoma, gastrointestinal stromal tumor, bladder cancer, and lung cancer [69–72]. CRC lineage-specific components provide information on tumor origin and enable selective dependence on tumor oncogenicity. Furthermore, disrupting the CRC in tumor cells via pharmacological or genetic suppression significantly reduces their tumorigenicity and malignant features [73]. Hence, the mechanism underlying CRC effects may lead to oncogenic addiction, suggesting a new approach to cancer therapy.

A panel of autoregulated master TFs leads a CRC based on SEs, which determine the cell-type-specific condition and malignant phenotype of tumor cells. For example, the SEs of TP63, a lineage-specific master TF, are co-occupied with other CRC TFs (SOX2, KLF5, and TP63), and three SE constituents are required to transcribe TP63 as well as induce esophageal squamous cell carcinoma (ESCC) progression [74]. Knockdown of any TF destroyed this CRC program, decreasing the accessibility of hundreds of chromatin sites in ESCC cells. Furthermore, research has revealed that TGIF1, EHF, and ELF3 create a transcriptional regulatory system that depends on a SE and interferes with gene transcription in lung cancer [69]. Similarly, other CRC TF (HOXB cluster genes and FOSL1)-mediated oncogenic transcription programs are involved in osteosarcoma tumor phenotypes [75]. More interestingly, in esophageal adenocarcinoma (EAC), four primary TFs EHF, GATA6, KLF5, and ELF3 showed enhanced mutual expression by interacting with every SE. These master TFs have been frequently linked with SEs and create interrelated CRCs by interacting with SEs. Each TF in the transcriptional circuit is unique to the EAC cell type and functions to enhance EAC cell survival and growth [76]. Therefore, the CRC model may be successfully used to answer scientific questions about cell identity and cancer biology, demonstrating the importance of CRCs in human cancer.

## SE complexes in tumors

SE complexes constitute a class of complexes that bind to SEs and regulate their function. A SE complex includes mainly TFs at high density, HMEs, and SE cofactors, such as BET family proteins, mediator complexes, and CDKs. Some SE complex components are used as markers to identify SEs. Moreover, SE regions can bind SE complexes and RNA Pol ll, enabling the regulation of targeted oncogenes in different tumor types [77].

**Fig. 3:**
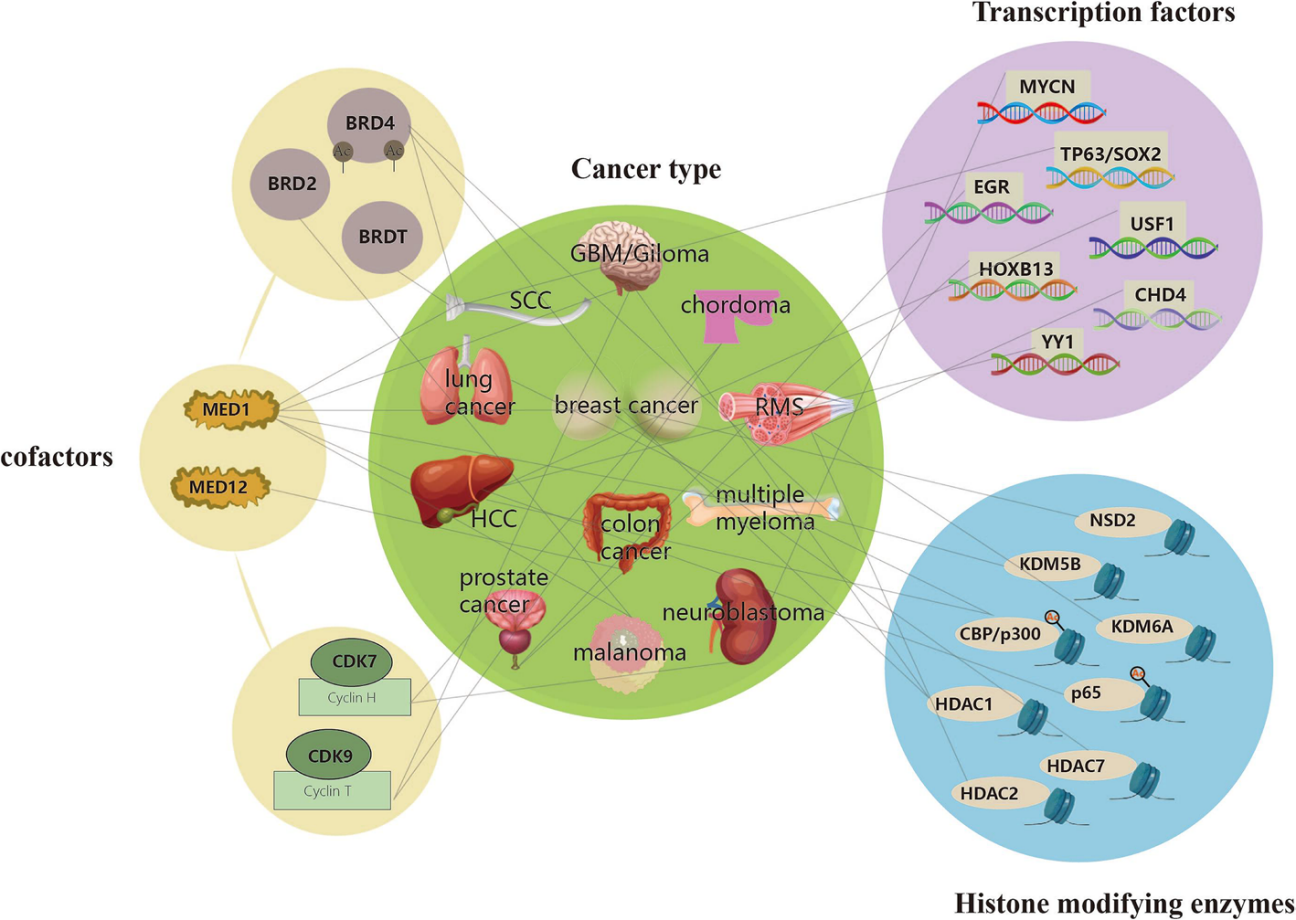
SE complexes, including the high density of TFs, HMEs, and SE-cofactors, such as BET family proteins, CDKs, and mediator complexes, are involved in the malignant phenotype of various tumors. This figure mainly proposed the correlation between SE complexes and tumor type. The green circles in the middle represent tumor types affected by SE complexes, other circles represent SE complexes, purple circles represent TF, blue represents HMEs, and yellow represents cofactors. For example, TF MYCN, ERG, HOXB13, USF1, TP63/SOX2, YY1 and CHD4 mediate SE, thereby affecting neuroblastoma, prostate cancer, hepatocellular carcinoma, squamous cell carcinoma, and rhabdomyosarcoma. HME NSD2, KDM6A, KDM5B, CBP/p300, P65, HDAC1, HDAC2, HDAC7 mediate SE, thereby affecting Multiple myeloma, rhabdomyosarcoma, breast cancer, hepatocellular carcinoma, glioblastoma. SE cofactors BRD2, BRDT, BRD4, CDK7, CDK9, MED1, MED12 mediate SE, thereby affecting Melanoma, squamous cell carcinomas, Multiple myeloma, neuroblastoma, chordoma, breast cancer, prostate cancer, giloma and colon cancer. ( GBM = Glioblastoma, HCC = Hepatocellular carcinoma, HMEs = Histone modifying enzymes, RMS = Rhabdomyosarcoma, SCC = Squamous cell carcinoma, TF = Transcription factor.)

This process underlying SE complex action is sequential. First, histone acetylating transferases such as p300/CBP are recruited by TFs, promoting nonhistone protein and nucleosome acetylation [78]. Second, BET proteins interact with hyperacetylated histone sites across chromatin, establishing transcriptionally active regulatory regions because of their increased affinity for proteins with many acetylated residues [78]. The establishment of synergistic high-density and cooperative transcriptional complexes at SEs is facilitated by BRD4 and mediator complexes, thereby altering the chromatin structure, dynamics, and function. Finally, the positive transcription elongation factor-b (P-TEFb) complex and CDK7 work in concert to release RNA Pol II and activate transcription. The P-TEFb complex phosphorylates the RNA Pol II CTD at serine 2, which activates its active transcribing form [79]. Further research into SE complexes will contribute to the development of antitumor drugs, offering additional treatments for many cancers that are currently incurable (Fig. 3).

### TFs

TFs, often referred to as trans-acting factors, bind to enhancers, SEs, or silencers and control the efficiency by which target genes are transcribed [80]. In 2013, SEs were first identified in ESCs because o ESC-specific TFs such as SOX2, NANOG, and OCT4 were found to be enriched at certain sites [2]. Currently, TFs are widely used to assist in identifying SEs in various diseases, including cancers. Moreover, SEs are commonly distinguished by the enrichment of many TFs that directly or indirectly bind to them and regulate their function.

Many TFs can directly bind to SEs to promote tumor progression. As early as 2014, research showed that the TF MYCN upregulated the active transcriptional program of neuroblastoma cell oncogenes by promoting SE function [39]. Other MYC family members can also use SEs as proliferation-controlling TFs, amplifying their transcriptional effects. For example, H3K27ac complementary nascent transcription and chromatin topology, a protein-centric analysis of chromatin conformation, led to the identification of multiple SEs 400–600 kb downstream of the MYC promoter that promoted primary effusion lymphoma cell growth and MYC expression [81]. Similarly, SE looped to the MYB promoter when the MYB protein bound to them, forming a positive feedback loop that maintained the expression of this master regulator expressed in ACC [41]. Ceribelli et al. announced that the E-box TF TCF4 directly binds to SEs to maintain the gene expression of blastic plasmacytoid dendritic cell neoplasm (BPDCN) [82]. Another group found that ERG is a TF in a SE that promotes the malignant development of PCa mainly by directly regulating lineage-specific enhancers and SEs [54]. Additionally, acetylated HOXB13 promotes the castration-resistant prostate cancer (CRPC) target genes FOLH1 and ACK1 and is abundant in tumor-specific SEs in PCa [83]. According to a recent study, the TF USF1, a SE component, drove the expression of the long noncoding RNA (lncRNA) FASRL in hepatocellular carcinoma (HCC) [84].

Moreover, interacting TFs combined with SEs promote tumor progression. Some evidence points to protein/nucleotide complexes, including TP63/SOX2, occupying the SEs of EGFR and promoting EGFR transcription in SCCs. The colocalization of the master TFs TP63 and SOX2 is a more common mechanism than occupancy by either TF, and this cobinding enrichment is specific to SEs. Notably, transcriptionally bound transcripts showed increased expression in the transcription phase when a SE is co-occupied by both TFs, indicating a particularly vital functional interaction between TP63 and SOX2 with SE elements [85]. Interestingly, TFs can recruit additional SE regulators to contribute to tumor progression. Recent studies revealed that YY1-p65-p300 enhanced the expression of QKI in HCC tumorigenesis [86]. Furthermore, in fusion-positive rhabdomyosarcoma, CHD4/NuRD colocalized with P3F at a SE, allowing tumor cell maintenance and survival. In the absence of CHD4, the SE lost its accessibility to DNA, causing tumor cell death. Given its role as a SE regulator, CHD4 may be a new potential target for cancer therapy [87].

Targeting oncogenic TFs in cancer, such as PML-RARA, has been shown to have significant therapeutic benefits when their application is practical [88]. Oncogenic TFs are very difficult to destroy with drugs. TF-directed drugs have been identified and optimized using novel platforms for discovery chemistry. Other potential strategies include methods for nimbly transporting biomolecules across cell membranes and, perhaps, the development of a whole new classes of medicines. One approach to achieving therapeutic aims is the use of a broadly usable chemical method for drug-induced degradation of specific proteins.

### Histone regulators

Epigenetic aberrations are considered critical initiators of tumors [89]. Histone modifications, known as epigenetic changes, are essential for tumor development and growth. There are many forms of histone modifications, including acetylation, methylation, phosphorylation, ubiquitination, and ADP ribosylation of histone ends, and these modifications can affect the transcriptional activity of genes. H3K4me1 and H3K27ac are two of the most typical histone modifications. Histone methylation and acetylation is mediated mainly in histone N-terminal tails and can affect the transcription of genes. Histone acetylation is mainly associated with gene activation, while methylation depends on the histone position and previous modification state and is associated with repression or activation [90]. Chromatin changes induced by chromatin compaction by histone writers (histone methyltransferases and histone acetyltransferases) and erasers (histone demethylases and histone deacetylases (HDACs)) regulate critical transcriptional pathways involved in tumor cell differentiation [91]. HMEs are recruited by TFs, which promote BET binding and interact with BETs. The enrichment of these histone modifications in a certain cell at a specific locus or genome-wide is identified by ChIP-based methods using antibodies against site-specific modifications to identify the presence of SEs.

SE-mediated histone lysine methyltransferase and histone lysine demethylase play important roles in tumorigenesis. For instance, the histone chaperone HJURP was aberrantly overexpressed in t(4;14)-positive MM due to its transcriptional activation mediated by a distal SE that had been induced by the histone lysine methyltransferase NSD2 [92]. Furthermore, a decrease in H3K4me1 in the FGFR4 SE has been associated with increased mRNA levels of KDM5B (a histone lysine demethylase) in alveolar rhabdomyosarcoma cells [93]. KDM6A, an X chromosome-linked histone lysine demethylase, has been reported to be frequently mutated in many tumor types, including breast and bladder cancer. The deletion of KDM6A activated SE-regulated genes that drive squamous cell differentiation and metastasis [94].

SE-mediated histone acetylation transferases and HDACs also play important roles in tumorigenesis. In the CBP/p300 complex, the main histone acetylation transferase in SEs, EP300 is a crucial component [95]. EP300 can directly regulate gene transcription by acetylating histones at gene SEs, suggesting that EP300 is necessary for gene transcription driven by SEs [96]. As described above, the YY1-p65-p300 complex binds to SEs and accelerates the function of QKI in HCC cells [86]. Another novel example of SE hijacking involves upregulated LINC01977, which promoted the acquisition of the malignant LUAD phenotype. Mechanistically, SE-driven LINC01977 enhances the building of the SMAD3/CBP/P300 complex and activates ZEB1 [97]. Furthermore, HDAC contributes significantly to chromosomal structural alterations and transcriptional repression through its deacetylase activity [98]. HDAC1, a class I HDAC family member, is associated with transcriptional control through its deacetylase activity. DNA-binding TFs reportedly recruited HDAC1 to form repressor multiprotein complexes with REST corepressor 1, nucleosome-remodeling deacetylase (NuRD), and Sin3 [99]. SE complexes with HDACs suppress histones in areas with cis-regulatory elements via H3K27ac deacetylation. For instance, the downregulation of the RET finger protein/HDAC1 complex increased the availability of temozolomide in glioblastoma by altering histone changes that affected cell division, apoptosis, and the cell cycle [100]. Ninety-nine percent of SEs are associated with HDAC2 binding, and HDAC2 shares SE-binding sites with core regulatory (CR) TFs. HDAC2 binds to the myogenic E-box and is loaded asymmetrically on enhancers of CR TFs in rhabdomyosarcoma cells [101]. Another published study revealed that HDAC7 was downregulated due to the downregulation of HDAC1/3, which was accompanied by a decline in H3K27ac abundance at a TSS and SEs, and was critical for maintaining cancer stem cells (CSCs) in breast cancer [102].

### SE cofactors

TF dysregulation in combination with SEs causes significant changes in the gene expression program in cancer cells. TFs can be considered regulatory elements that do not directly bind to DNA in a sequence-specific manner but send signals to RNA PoI II through transcription cofactors. BET family proteins, the mediator complex, and CDKs, which are attracted to SE-promoter areas by TFs in the context of activated transcription, are examples of this class of transcriptional signaling proteins. They can bind directly or indirectly to SEs, thereby affecting tumor progression.

#### BET family proteins

The BET family of bromodomain proteins (BRDs) consists of four members: BRD2, BRD3, BRDT, and BRD4. Typically, they regulate gene transcription by binding to SE regions and are expressed in several cancers.

BRD2 proteins are among the many kinds of bromodomain-containing proteins widely expressed and essential for cell cycle regulation in healthy mammalian cells. Previous studies found that BRD2, a cofactor of STAT5, binds strongly to c-Myc SE regions, which has been recently reported to be essential for leukemia maintenance [103]. Similarly, BRD2 binds to the AMIGO2 promoter and SE configuration, which is essential for melanoma cell survival [104]. BRD3 functions as a molecular sensor of cellular activity and is critical to the overall concentration of BET family proteins via its role in the compensatory regulation of BET protein levels. For example, BRD3 is attracted to a subset of estrogen receptor alpha binding sites (ERBSs) enriched with functional enhancer characteristics, found in groups of ERBSs that probably serve as "SEs" and are connected to genes with high E2-responsiveness. Furthermore, the clusters of ERBSs enriched in BRD3 that were discovered in this study may be classified as SEs that collectively activate essential ER-regulated genes [105]. BRDT, as a particular type of BET family protein, is expressed only in the testis. Wang et al. discovered that BRDT interacted with Np63 and colocalized with it to activate a distinct transcriptional program by targeting a SE to promote the migration of ESCC cells [106]. These findings reveal critical functions of BRD2, BRD3, and BRDT in tumors and suggest a novel treatment strategy.

BRD4 is a crucial epigenetic modification and transcription regulator consisting of two tandem bromine domains (BD1, BD2) [107]. BRD4 reads the histone code and accumulates on transcription- and hyperacetylation-prone chromatin sites (SEs and promoters). After attaching to acetylated chromatin sites, BRD4 activates the mediator complex, promotes RNA Pol II activity, and initiates transcription, and elongation factors are assembled at these sties, which are their nucleation centers [108]. For example, BRD4, MEDs, and P-TEFb heavily accumulate on SEs in MM cells [78]. BRD4 and the mediator complex work together to establish the transcriptional complex machine that alters the transcription of SE-related target genes. In addition, BRD4 forms SEs at cancer stemness genes by carefully colocalizing with MED1 and p65 in SCC cells. Although neither p65 nor MED1 is required for BRD4 binding in SEs, BRD4 recruits p65 and MED1 to facilitate SE development [109].

BRD4 has also been shown to interact with many chromatin-remodeling proteins. In breast cancer, BRD4 and the lysine-specific demethylase 1/nucleosome-remodeling and deacetylase complex interact with each other and are colocalized at SEs. SEs driven by the BRD4/LSD1/NuRD complex significantly affect many well-characterized cellular signaling pathways essential for the growth, survival, and homeostasis of cancer cells. The BRD4/LSD1/NuRD complex is closely associated with SEs and can inhibit the activation of a set of genes, such as drug resistance genes [110]. Another research group found that methylated BAF155 shares genome binding sites with BRD4 and H3K27ac, and methylated BAF155 increased tumor metastasis by attracting BRD4 and activating SE-dependent oncogenes in breast cancer [111]. Due to its role in the formation of SEs and control of oncogene expression, BRD4 is a well-established factor in cancer cells. However, the importance of BRD4 in cancer goes beyond regulating transcription as this protein is a custodian of genomic integrity. In fact, it has been shown that BRD4 plays a nontranscriptional role in regulating DNA damage checkpoint activation and repair as well as telomere preservation, shedding new light on’the many roles of BRD4 and providing fresh ideas for the application of BET inhibitors in cancer.

#### The Mediator complex

The Mediator complex is a multisubunit complex comprising 25–30 subunits that directly pair with the various subunits to control the activity of several TFs [112]. It controls gene transcription by functioning a functional link between the RNA Pol II-associated basal transcription machinery and TFs unique to certain genes. MED1, also referred to as PBP, DRIP205, and TRAP220, is an essential part of the mediator complex because it targets and anchors the complex to various nuclear receptors and cell type-specific TFs [113]. The SE complex known as the component MED1 has been found to play an essential role in MM, melanoma, breast cancer, PCa, and glioma [114–118]. In addition, either MED12 or MED13/MED13L can bind to SEs, and both MED12 and MED13/MED13L are required for SE-related gene expression in stem cells [112]. A disproportionate decrease in SE gene expression results from deleting either MED12 or MED13/MED13L. For example, in colon cancer cells, the abrogation of MED12 or MED13/MED13L reduced the expression of SE genes such as MYC, resulting in reduced cell proliferation [119].

MED1 is a component of the mediator complex, and MED1 can directly bind SEs in noncancerous and cancerous tissues. For example, MED1 can directly bind to SEs of several oncogenes to inhibit the proliferation and colony formation of HCC cells. Additionally, the upregulation of MED1 is related to a poor prognosis in HCC patients [120]. Interestingly, in a study MED1 was located in the SEs of an active gene in the MM genome of control cells. In MM cells treated with the SE inhibitor JQ1, the MED1 levels were decreased, mainly at enhancers, with maximum loss at SEs [114]. MED1 also interacts with TFs to bind to SEs. Zhou et al. reported that SEs co-occupied by INO80 and MED1 were more abundant in genes specifically expressed in melanoma than SEs occupied by MED1 alone, which may provide new insights into melanoma tumorigenesis [115]. Recently, we reported that MED1 and Myc interact to regulate SEs of TMEM44-AS1 and promote their glioma cell-specific transcriptional activation [116]. Sena et al. reported that the interaction of the Androgen Receptor (AR) and mediator complexes in SEs might contribute to advanced PCa [121]. A further study observed co-occupancy of AR and MED1 on chromatin, especially when SEs were enriched in CRPC cells [117]. Interestingly, in breast cancers in which the MED1-bound estrogen receptor shares a SE, MED1 acts as a promoter for SE interactions with neighboring enhancers [118].

#### CDKs

CDKs are threonine/serine kinases that are essential for transcription and cell cycle control [122]. Cyclin overexpression or hyperactivation, inhibition of CDKI activity, and continuous activation of upstream division signaling all change the activity of CDKs in tumor cells [123]. Moreover, CDKs, among SE complexes, can influence tumor progression. The CTD of Pol II is phosphorylated by transcriptional CDKs, particularly CDK7, a subunit of TFIIH, and CDK9, a subunit of pTEFb, promoting effective transcriptional start, pause, release, and elongation. When in an active state, the P-TEFb complex interacts with cofactors and TFs, phosphorylates factors that cause transcription pause, and phosphorylates the Ser2 residue in the CTD of RNA-Pol II to activate transcription [124].

Because the activity of CDKs is necessary for cell division, CDKs are better therapy targets for tumors and other disorders. A previous study showed that the SE regulator CDK7 inhibitor THZ1 caused significant tumor regression in MYCN-amplified neuroblastoma cells and affected RUNX1 transcription in Jurkat T-ALL cells [125]. Similarly, nasopharyngeal carcinoma and Ewing sarcoma cells were highly sensitive to THZ1 [126, 127]. Additionally, CDK9 has been found in promoter-distal intergenic regions, including SEs. eRNA transcription is necessary for the expression of SE-related genes and is also thought to be regulated by CDK9 [5]. Treatment with the CDK9 inhibitor flavopiridol reduced eRNA transcription elongation and supported the functional role of CDK9 with respect to SEs [128]. Moreover, chordoma cell proliferation was effectively suppressed by CDK inhibitors that target CDK7/12/13 and CDK9 [129]. Together, these studies suggest that CDKs may be potential therapeutic targets for cancer. In the future, developing clinically useful inhibitors of CDKs will require a further mechanistic understanding of the exact functions that CDKs play in conjunction with SE complexes and transcriptional addiction in cancer pathogenesis.

## The role of super-enhancer-driven oncogenes in tumors

**Fig. 4:**
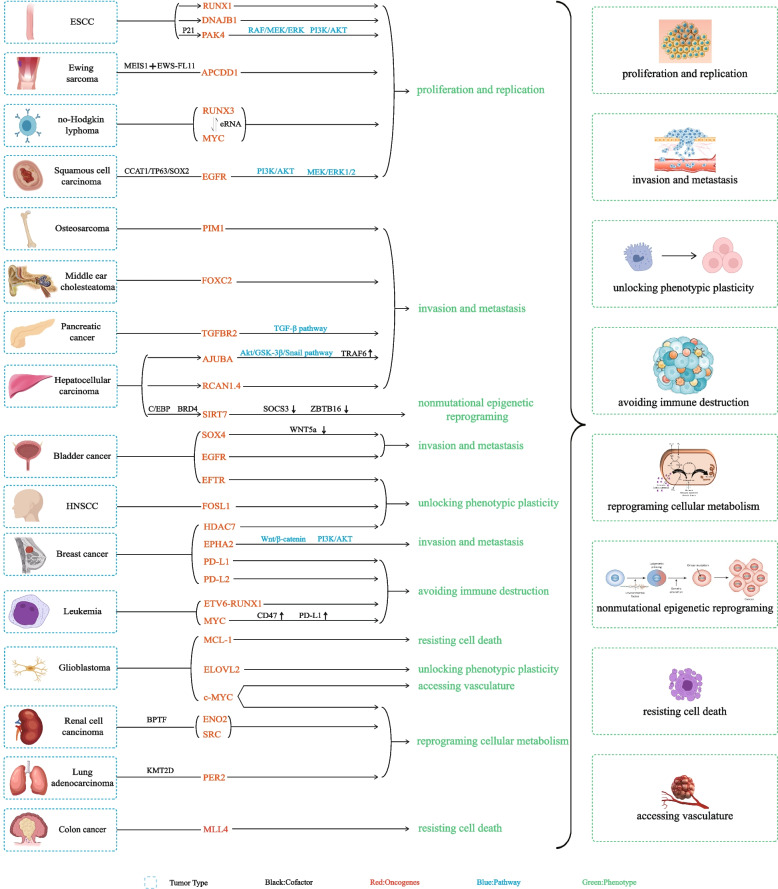
The role of SEs-driven oncogenes in tumors. During tumor development, SE-associated oncogenes affects many aspects of it, including sustaining proliferative signaling and enabling replicative immortality, evading activating invasion and metastasis, avoid immune destruction and promoting inflammation, reprogramming cellular metabolism, nonmutational epigenetic reprogramming, unlocking phenotypic plasticity, resisting cell death and inducing/accessing vasculature. This figure mainly proposes the roles and mechanism of SEs-driven oncogenes, as well as the corrrsponding relationship with tumor types. Black words represent cofactors. Red words represent oncogenes. Bule words represent pathways. Green words and the contents in green boxes represent phenotypes. The contents in the blue boxes indicate tumor types. The “↓” means down-regulation and the “↑” means up-regulation. The “⇋” means interaction between the two. The” + ” means “and”. (ESCC = esophageal squamous cell carcinoma, HNSCC = Head and neck squamous cell carcinoma.)

As seen in many malignancies, the expression levels of SE-related genes are often significantly different than those controlled by TEs, suggesting that SE-related genes may be potential markers and therapeutic targets. During tumor development, via SE acquisition, tumor cells may show markedly different expression of multiple oncogenes. Therefore, SE-related genes can affect tumors by sustaining cell proliferation-related signaling and enabling replicative immortality, evading immune surveillance, activating invasion and metastasis, avoiding immune defenses and promoting inflammation, reprogramming cellular metabolism, inducing nonmutational epigenetic reprogramming, unlocking phenotypic plasticity, resisting cell death and inducing angiogenesis/accessing the vasculature [130] (Fig. 4).

### Super-enhancer-driven oncogenes in cell proliferation and replication

Normal tissues achieve homeostasis via cells through the regulation of proliferation and replication signaling. However, cancer cells can escape this regulation and proliferate indefinitely, many through the coordinated action of SE-associated oncogenes. Hosoi et al. reported that the SEs of MYC and RUNX3 may functionally interact through their eRNAs to promote non-Hodgkin lymphoma cell proliferation [131]. Lin et al. found that EWS-FLI1 and MEIS1 were recruited to APCDD1 SE elements, increasing the transcription and proliferation of APCDD1, which exerted a significant pro-survival effect [127]. Although increasing evidence supports the proposition that these SE-related oncogenes play crucial roles in cancer cells, whether they can be used in clinical cancer treatment is unknown, and further research is warranted. SE-related oncogenes can regulate the proliferation and replication of cancer cells by influencing signaling pathways. For example, in ESCC, several SE-related oncogenes influence tumor proliferation, such as RUNX1, DNAJB1, and PAK4. Overexpression of PAK4 can activate tumor signaling pathways, such as the PI3K/AKT and RAF/MEK/ERK pathways, while low expression of PAK4 can inhibit the growth of ESCC cells. At the same time, DNAJB1 is highly expressed, and together with RUNX1, affects proliferation [132]. Additionally, SE-driven EGFR activates EGFR expression, promotes the PI3K/AKT and MEK/ERK1/2 signaling pathways, and increases SCC cell proliferation in vivo and in vitro [85].

### Super-enhancer-driven oncogenes regulate cell death resistance and induce angiogenesis

In addition, to protect tumor growth, programs that enable cells to circumvent death play equally vital roles in tumor tissue, as they prevent apoptosis, autophagy, or necrosis. Many of these processes rely on tumor oncogene activity. It has been verified that Mcl-1, an antiapoptotic member of the Bcl-2 family, whose SEs were identified in glioblastoma and mesothelioma, acts as a key molecule in apoptosis control, promoting cell survival [133]. A recent study reported that MLL4 knockdown inhibited RNA-induced silencing complex and DNA methyltransferase expression in B16F10 and MC38 cells by reducing the enhancer/SE, causing transcriptional reactivation of the dsRNA-interferon response and pyroptosis induced by gasdermin D, respectively [134]. Moreover, tumor angiogenesis is characterized by the growth of a blood vessel network that provides a tumor a favorable microenvironment with nutrients and oxygen that enable optimal growth [135]. Studies have shown that c-Myc, a SE-related gene, promotes glioblastoma angiogenesis [102, 136]. This study direction, however, is worthy of additional attention due to the necessity for thorough knowledge of the mechanism underlying SE-related oncogenes in tumor angiogenesis.

### Super-enhancer-driven oncogenes mediate cell invasion and metastasis

Cancer cells show the propensity to invade and metastasize, which allows them to exit the initial tumor mass and colonize in new tissues throughout the body. Recent research has demonstrated that SE-related oncogenes are crucial in this process. For example, the SE-driven oncogenes SOX4 and EGFR participate in cell migration and invasion in bladder cancer. The SE-related oncogene SOX4 can either indirectly or directly suppress WNT5a levels, and WNT5a most likely protects bladder cancer cells from invasion [137, 138]. Similarly, the SE-driven oncogene EGFR contributes to cancer cell migration and invasion, promotes bladder cancer development and progression, and shows prognostic value [139, 140]. In addition, SE-driven oncogene PIM1 knockdown has been shown to substantially inhibit osteosarcoma cell migration and invasion [141, 142]. Recent studies have shown that Epha2-SE deletion leads to decreased EphA2 expression, which inhibits Wnt/β-catenin and PI3K/AKT pathway activation, thereby inhibiting the invasion and migration of cancer cells, including MCF-7 cells, HCT-116 cells, and HeLa cells [143]. Notably, some SE-associated tumor suppressor genes play essential roles in metastasis. For instance, overexpression of the SE-driven tumor suppressor gene RCAN1.4 in HCC cells reduces xenograft tumor metastasis by decreasing calcineurin activity and NFAT1 nuclear translocation [27, 144]. Therefore, it is crucial to continue researching the use of RCAN1.4 in HCC.

The development of altered epithelial cell invasion, apoptosis resistance, and dissemination is related to epithelial-mesenchymal transition (EMT) [145, 146]. The EMT is now widely believed to be associated with tumor development and spread as it can increase tumor migration, aggressiveness, and other cancer characteristics [147]. EMT is also regulated by some SE-related oncogenes in tumors, including cholesteatoma, HCC, and pancreatic cancer. For instance, the SE-associated oncogene FOXC2 drives the EMT by upregulating snail expression and downregulating E-cadherin expression in cholesteatoma [148]. Zhang et al. reported that SE-driven AJUBA overexpression attracted tumor necrosis factor receptor-associated factor 6 and promoted the EMT in HCC cells by activating the Akt/GSK-3/Snail pathway [149]. Furthermore, SE-driven TGFBR2 has been shown to involve the TGF-β pathway in pancreatic cancer cells, thereby inducing the EMT as tumor promoters. SgRNA-mediated deletion of this SE significantly reduced TGFBR2 expression and affected the TGF-induced EMT in smad4 ( +) pancreatic cell lines [150].

### Super-enhancer-driven oncogenes and establishing phenotypic plasticity

The capacity of a single genotype to display several phenotypes in various settings, or phenotypic plasticity, declines with cell development and differentiation and, in most situations, hinders cells proliferation. Thus, there are several pathways that establish phenotypic plasticity in cancer cells, including dedifferentiation, blocking differentiation, and transdifferentiation. Recent studies suggest that these processes may be regulated by SE-related oncogenes. For instance, EGFR, previously associated with SEs, is a crucial factor in bladder cancer cell dedifferentiation expressed in the basal layer and associated with a less differentiated state of normal urothelial cells [140, 151].

CSCs, which are maintained in an undifferentiated state, also play various roles in this process, maintaining the advancement of disease and interacting with their environment to leverage important survival variables [152, 153]. Gimple et al. reported that ELOVL2 was identified as a glioma stem cell-specific SE-associated gene that stimulated the production of LC-PUFAs, which is required to preserve stem-cell membrane architecture and promote EGFR signal transduction [154]. In addition, HDAC7 inactivation inhibited the acquisition of a CSC phenotype in the breast by downregulating the enrichment of H3K37ac in several SE-associated oncogenes, including CDKN1B, c-MYC, SLUG, XBP1, HDAC7, BCL-XL, VDR, SMAD3, CD44, and VEGFA [102]. Another study showed that the expression of the SE-associated oncogene FOSL1 was higher in head and neck squamous cell carcinoma (HNSCC) CSCs than in non-CSCs, which may be related to the preservation of CSC-like characteristics, such as self-renewal, tumorigenesis, and metastasis [155].

### Super-enhancer-driven oncogenes and tumor immune destruction

According to immunosurveillance theory, a constantly alert immune system constantly monitors cells and tissues, identifying and destroys many early cancer cells [156]. However, tumor tissue typically lacks immunological checkpoints and continues to grow. The SE generated by the fusion gene ETV6-RUNX1 is regarded as a critical marker for CD19 + /CD20 + cells at a late stage of B-cell differentiation and is related to B-cell maturation in acute leukemia [157]. Furthermore, the expression of immunological checkpoints, including inhibitory and stimulatory checkpoints, is also influenced by SE-associated oncogenes. For instance, MYC, which has been previously associated with SE, increases the expression of the innate immunological checkpoints cluster of differentiation 47 and programmed death-ligand 1 (PD-L1) in acute leukemia and HCC cells by directly interacting with the promoters of these two genes [102, 158]. In addition, a study on PD-L1/PD-L2 confirmed the presence of a SE in breast cancer that enhances the expression of PD-L1 and PD-L2, thereby promoting tumor immune escape [159].

### Super-enhancer-driven oncogenes involved in reprogramming cellular metabolism

Reprogramming of metabolism in tumor cells involves the reprogramming of glucose metabolism in the presence of oxygen to regulate energy production; this process is also known as aerobic glycolysis. It enables tumor cells to remain viable and undergo unlimited proliferation under extreme microenvironmental conditions and promotes tumor cell progression and metastasis. Research has shown that a SE composed of BPTF was shown to activate the oncogenes ENO2 and SRC as an effective switch for manipulating glycolysis in renal cell carcinoma, leading to glycolytic reprogramming [160]. In addition to glucose metabolism, SE-driven oncogenes regulate other metabolic pathways in tumor cells. Tateishi et al. reported that the SE-associated gene c-MYC regulated nucleotide biosynthesis and single-carbon metabolism in glioblastomas [102, 161]. Similarly, Wang et al. found that both cholesterol synthesis and fatty acids were regulated by c-MYC in glioblastoma stem-like cells [162]. Alam et al. reported that KMT2D upregulated the SEs of PER2 and promotes PER2 expression, thereby inhibiting glycolysis by decreasing the expression of glycolysis genes, including Pgk1, Cdk, Pgam1, Gapdh, Eno1 and Ldha, in LUAD [163]. These studies suggest that SE-associated gene-mediated metabolic pathways are reprogrammed to meet bioenergy, biosynthesis, and redox requirements, which has important implications for tumor cell survival.

### Super-enhancer-driven oncogenes and nonmutational epigenetic reprogramming

Nonmutational epigenetic reprogramming mainly involves epigenetically mediated changes in gene expression [130]. Several recent studies have suggested that the abnormal physical characteristics of the tumor microenvironment are closely related to the heterogeneity of epigenetic regulation. Changes in the SE components of some oncogenes affect the epigenetic inheritance of cancer cells. Histone acetyltransferase C/EBP occupies a SIRT7 SE, recruits BRD4 through H3K27 acetylation to upregulate SIRT7, and then epigenetically silences several metabolic regulators, including SOCS3 and ZBTB16, in HCC cells. Furthermore, H3K18 is deacetylated across the genome by SIRT7 SE activation, which functions in concert with the H3K27 methyltransferase EZH2 to epigenetically silence genes [164]. An increasing body of evidence suggests that similar epigenetic changes might contribute to the development of tumor growth and progression. Additional epigenetic modifications, including DNA and RNA methylation, are involved in super enhancer-driven mediation-based malignant phenotypes of tumors.

## The roles of SE-associated ncRNAs in tumors

With the development of high-throughput techniques, we have found that protein-coding genes account for less than 2% of the human genome, and most nucleotide sequences produce ncRNA with no protein-coding activity [165]. With a threshold of 200 nucleotides, ncRNAs are often classified into short ncRNAs and lncRNAs. By comparing their morphologies, circular RNAs (circRNAs) and linear lncRNAs can be distinguished from each other. LncRNAs, circRNAs, and microRNAs (miRNAs) are other categories of ncRNAs [166]. It has been reported that SEs indirectly control biological processes by activating the expression of ncRNAs in addition to genes encoded by exons. SE-derived ncRNA is involved in biological processes in various cancers, including unchecked proliferation, apoptosis, invasion and metastasis, chemical resistance, and tumor inflammation [167]. Therefore, understanding the potential mechanism of SE-related ncRNAs in tumorigenesis may inspire a new approach for early diagnosis and targeted therapy of tumors.

### SE-associated lncRNAs

**Fig. 5:**
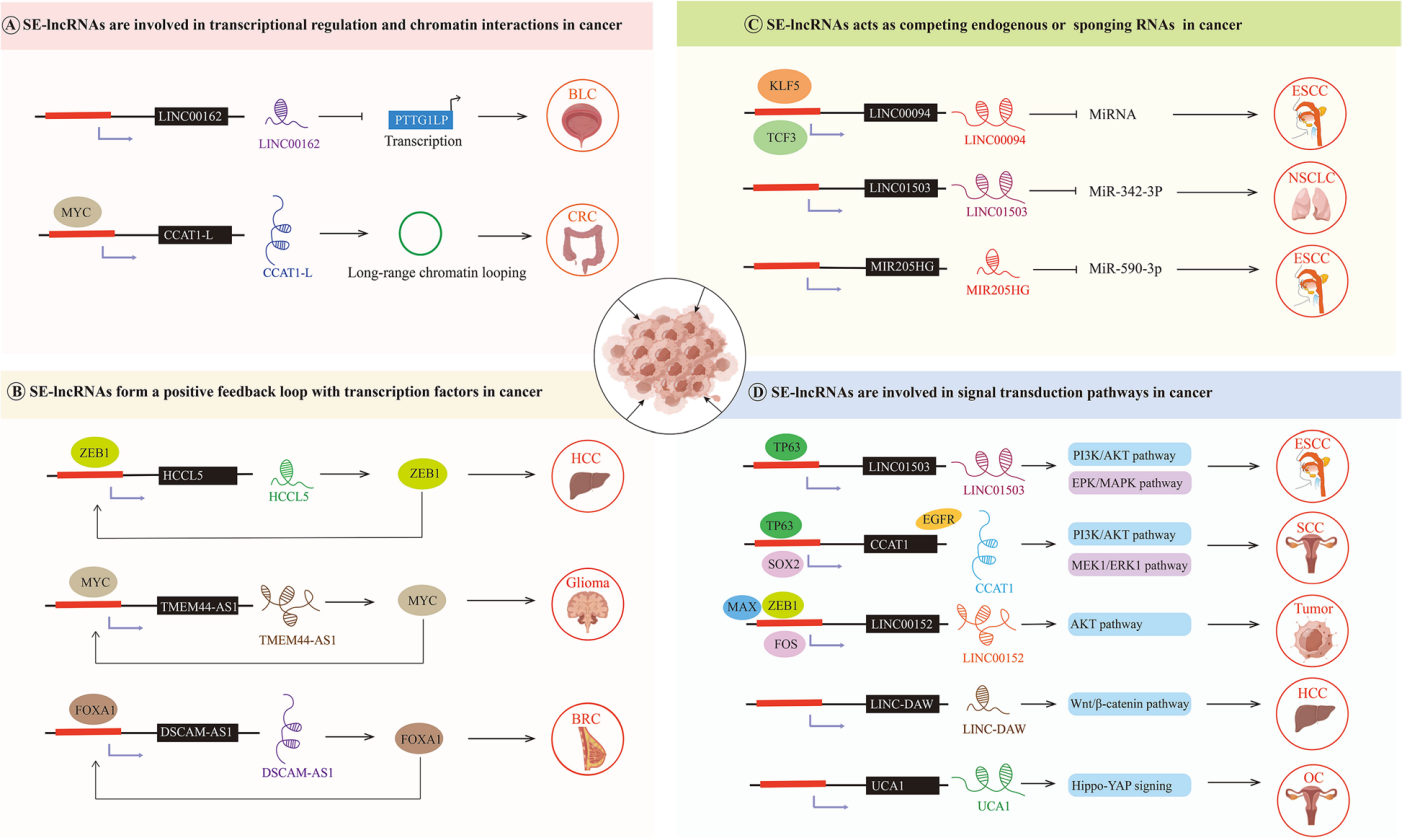
SE-associated lncRNA in tumors. Red rectangles represent SEs, black rectangles represent lncRNA, acute-angle arrows represent facilitation, and right-angle arrows represent repression in the figure. (A)SE-lncRNAs are involved in transcriptional regulation and chromatin interactions in cancer. SE-LINC00162 inhibits the transcription of PTTG1IP, hence reducing the expression of PTTG1IP and promoting the proliferation of bladder cancer cells. SE-CCAT1-L, located within a SE and close to MYC, promotes long-range chromatin looping in human colorectal cancers. (B)SE-lncRNAs form a positive feedback loop with TFs in cancer. HCCL5 is overexpressed in human HCC tissues and is regulated by ZEB1 transcription through a SE, while increased HCCL5 exacerbates the EMT phenotype by inducing ZEB1 expression, which creates positive feedback. LncRNA DSCAM-AS1 can interact with YBX1, controls the expression of FOXA1, and advances breast cancer. FOXA1-driven SEs can also transcribe and activate lncRNA DSCAM-AS1 to create a positive feedback loop. (C)SE-lncRNAs act as competing endogenous or Sponging RNAs in cancer. In non-small cell lung cancer samples, SE-lncRNA LINC01503 may competitively bind LASP1 with miR-342-3p. MIR205HG depletes endogenous miR-590-3p leading to increased YAP, cdk1, and cyclin B protein expression in Head and Neck Squamous Cell Carcinoma. (D)SE-lncRNAs are involved in signal transduction pathways in cancer. LncRNA UCA1 expression is positively correlated with SEs and has been identified as a regulator of the Hippo-YAP signaling pathway, highlighting the role of the UCA1-AMOT-YAP signaling axis in ovarian cancer progression. (BLC = Bladder cancer, BRC = Breast cancer, CRC = colorectal cancer, ESCC = esophageal squamous cell carcinoma, HCC = Hepatocellular carcinoma, NSCLC = Non-small cell lung cancer, SCC = Squamous cell carcinoma, OC = Ovarian cancer.)

Over the past 10 years, it has become increasingly obvious that the genomes of many species undergo extensive transcription, producing large quantities of lncRNAs [168]. LncRNAs are transcripts of more than 200 nucleotides found in the cytoplasm and nucleus but are not translated into proteins. Many studies on lncRNAs have shown that they can be classified into many types based on their genomic location and context; these types include intergenic lncRNAs, intronic lncRNAs, bidirectional lncRNAs, and sense and antisense lncRNAs [169]. LncRNAs can affect gene expression through histone modification, transcriptional regulation, and post-transcriptional regulation, participating in almost all cell biological processes. LncRNAs functions primarily by acting as corepressors or coregulators, recruiting and interacting with proteins, acting as decoys, interacting with miRNAs, and acting as host genes for miRNAs. LncRNAs are also involved in tumor cell differentiation, proliferation, apoptosis, invasion, and metastasis, influencing the development of several cancers. Recent research has revealed that SEs influence the biological process of tumors by promoting lncRNA transcription in addition to directly driving the transcription of coding genes [167] (Fig. 5).

LncRNAs produced by SE generally come in two forms: eRNA and SE-derived lncRNAs. eRNA is generated by the self-transcription of SEs with a sequence length of 0.5–5 kb and can alternatively be classified as a lncRNA [170]. eRNAs influence gene expression through a variety of regulatory modes. For example, eRNAs generated from the TP53 SE region enhanced effective TP53 transcription and induced P53-dependent cell cycle arrest, indicating that the eRNA transcribed by TP53 SE plays an important role in inhibiting the proliferation of cancer cells [171]. The other type is an lncRNA, which is typically referred to as a SE-derived lncRNA. The promoter region of these lncRNAs is transcribed, and SEs control this process. SE-derived lncRNAs play an essential role in the inflammatory response, drug resistance, metastasis, and malignant proliferation of cancer. Mechanistically, the association of SE-lncRNAs with other cellular macromolecules, including RNAs, proteins, and DNAs, allows them to control various important cancer cell characteristics [167].

Notably, the biological activities of lncRNAs are in part determined by the cellular location of the molecule. Nuclear SE-lncRNAs are more functional in terms of transcriptional control and chromatin interactions. For example, SE-LINC00162 is chiefly located in the nucleus and is highly expressed in bladder cancer tissues and cells. SE-LINC00162 inhibits the transcription of PTTG1IP activated by THRAP3, thereby reducing the expression of PTTG1IP and promoting the proliferation of bladder cancer cells [172]. Moreover, it has been demonstrated that a lncRNA, CCAT1-L, located in a SE and close to MYC, is transcribed from 515 kb upstream of MYC in human colorectal tumors [173]. This lncRNA promotes long-range chromatin looping and participates in the control of MYC transcription. The structure of this chromatin ring changes during cancer development, altering the genetic instructions. Here, a lncRNA may function by interacting with other molecules to create a scaffold for a protein complex that connects an enhancer-like region and a coding gene promoter. Das et al. reported that Ang II stimulated VSMC proliferation and angiogenesis by inducing the transcription of SE-derived lnc-Ang383. However, whether Ang II/SE-derived lncRNAs contribute to tumor proliferation and angiogenesis remains to be further investigated [174] (Fig. 5A).

In contrast to nuclear SE-lncRNAs, cytoplasmic SE-lncRNAs can interact with TFs to form a positive feedback loop that promotes carcinogenesis. Peng et al. reported that HCCL5 was overexpressed in human HCC tissues and regulated by ZEB1 transcription through a SE, but when the HCCL5 level was increased, the EMT phenotype was acquired because it induced ZEB1 expression, which created a positive feedback mechanism [175]. Similarly, we previously reported that SE-associated TMEM44-AS1 is immediately linked to SerpinB3, activating EGR1/IL-6 signaling and Myc; Myc immediately attaches to the TMEM44-AS1 SE being transcriptionally activated, aggravating the growth of gliomas through a positive feedback loop established with TMEM44-AS1 [116]. Additionally, lncRNA DSCAM-AS1 interacts with YBX1, controls the expression of FOXA1 and estrogen receptor α, and advances breast cancer. FOXA1-driven SEs also transcribe and activate lncRNA DSCAM-AS1 to create a positive feedback loop [176] (Fig. 5B).

In contrast, cytoplasmic SE-lncRNAs affect post-translational modification via competing endogenous RNAs (ceRNAs) or by functioning as molecular sponges, which alter miRNAs that govern the expression of target genes. For instance, the SE-associated ce-lncRNA LINC00094 can be activated by the TFs KLF5 and TCF3 that bind to SE regions and significantly regulate the expression of cancer-related hallmark genes in ESCC cells [177]. In non-small cell lung cancer samples, the expression of SE-lncRNA LINC01503 and LIM and SH3 domain protein 1 (LASP1) was increased, while the expression of miR-342-3p was downregulated, suggesting that SE-lncRNA LINC01503 may competitively bind LASP1 with miR-342-3p [178]. More interestingly, MIR205HG depleted endogenous miR-590-3p, leading to increased YAP, cdk1, and cyclin B protein expression in HNSCC cells [179]. However, a comprehensive understanding of how SE-lncRNAs regulate gene expression is still lacking (Fig. 5C).

Furthermore, cytoplasmic SE-lncRNAs are involved in signal transduction pathways in cancer. The TP63-regulated SE-lncRNA LINC01503 activates the ERK/MAPK and PI3K/Akt signaling pathways and increases ESCC cell proliferation and invasion [178]. According to a recent study, the coactivation of SE-driven CCAT1 by TP63 and SOX2 regulated EGFR expression by binding to the SEs of EGFR, which activated the MEK/ERK1/2 and PI3K/AKT signaling pathways in SCC cells [85]. Moreover, SE-driven lncRNA-DAW is frequently increased in HCC and promotes HCC cell growth by mediating EZH2 degradation and activating the Wnt/beta-catenin pathway [180]. Xu et al. analyzed the regulatory network of LINC00152 in pancarcinoma and found that the TFs MAX, ZEB1, and FOS likely bound in the SE region upstream of LINC00152 and that reducing LINC00152 reduced the invasion and metastasis of breast cancer. LINC00152 regulates AKT signaling pathway activation, which is vital in tumor chemical resistance [181]. Another study revealed that lncRNA UCA1 expression was positively correlated with SEs and has been identified as a regulator of the Hippo-YAP signaling pathway, highlighting the role of the UCA1-AMOT-YAP signaling axis in ovarian cancer progression [182]. These studies suggest that SE-associated lncRNAs mediated by TFs can activate carcinogenic signaling pathways, affecting tumor proliferation, invasion, and chemical resistance. However, the evidence for using SE-lncRNAs as therapeutic cancer targets is insufficient, and further clinical validation is needed (Fig. 5D).

### SE-associated miRNAs

**Fig. 6:**
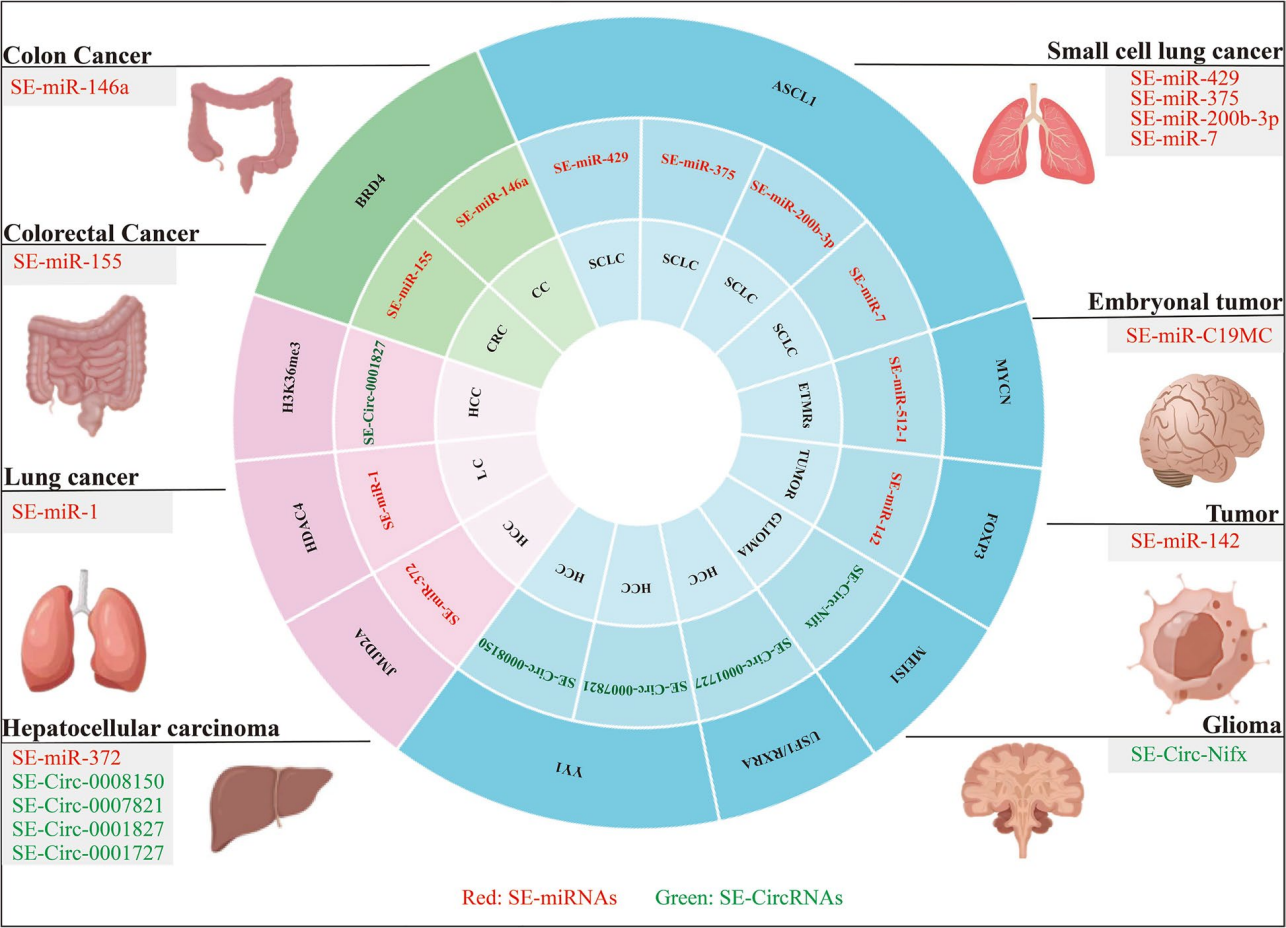
SE-associated miRNAs and SE-associated circRNAs mediated by TF and HMEs are implicated in the development of tumors. The circular outer ring represents the three SE complexes (Blue represents the TF, Pink stands for the HMEs, Green stands for the SE cofactors). The loop in the middle of the circle is the SE-associated miRNAs and SE-associated circRNAs regulated by the SE complex (Red word represents SE-associated miRNAs, Green word stands for the SE-associated circRNAs). The inner loop of the circle represents tumors affected by SE complexes that regulate SE-associated miRNAs and SE-associated circular RNAs. For instance, TF ASCL1 positively control SE-associated miRNAs (SE-miR-429, SE-miR-375, SE-miR-200b-3p, and SE-miR-7), which helps define molecular subtypes of small cell lung cancer. (CC = Colon cancer, CRC = Colorectal cancer, ETMRs = Embryonal tumors with multilayered rosettes, HCC = Hepatocellular carcinoma, HMEs = Histone modifying enzymes, LC = Lung cancer, SCLC = Small cell lung cancer, TF = Transcription factor.)

miRNA, a noncoding small RNA with a length of 18 ~ 23 nucleotides, affects post-transcriptional gene regulation [183]. With advancements in of miRNA research and the development of deep sequencing technology, increasing evidence shows that miRNA is an important bifunctional molecule. miRNA is located in the cytoplasm can mediate post-transcriptional gene silencing via sequence-specific inhibition of target mRNA translation. For instance, the earliest miRNA discovered, let-7 reduces the invasiveness and chemotherapy resistance of cancer by downregulating MYC, HMGA2, BLIMP1, or RAS family members, thereby inhibiting the development of tumors [184]. In contrast, when it is located in the nucleus, let-7 changes the chromatin state of the enhancer by combining the enhancer, thereby activating the transcription and expression of genes. In addition, hsa-miR-24–1, hsa-miR-3179, and hsa-miR-26a-1 are located in the nucleus and activate adjacent or remote genes by targeting enhancers [185]. These miRNAs showing gene activating functions in the nucleus are defined as nuclear-activating miRNAs [186]. Moreover, super-enhancer-associated miRNAs (SE-miRNAs) constitute a group of unique miRNAs transcribed from the genome region with the SE. Recent research has revealed that SE-miRNAs are closely related to several human diseases, including cancer. Based on the components of the SE complexes bound to the SE, SE-miRNAs can be classified into different categories (Fig. 6).

A TF is a critical component of SE complexes and mediates SE-related miRNAs in many tumors. SEs have been reported to drive cell type-specific miRNA biogenesis, and sets of miRNA genes have been identified for 86 different types of human tissue and cell samples. Four SE-associated miRNAs were identified in small-cell lung cancer cells, including miR-429, miR-375, miR-200b-3p, and miR-7. In addition, the TF ASCL1 positively controls these SE-associated miRNAs, and the regulation of target genes by SE-associated miRNAs may help distinguish small-cell lung cancer molecular subtypes [187]. Additionally, a large, chromatin-accessible region flanked by TTYH1-miR-512–1 fusion breakpoints contains a miR-512–1-associated enhancer element located 20.3 kb downstream of the genomic breakpoint, indicating that miR-512–1 is a SE-associated miRNA. SE-miR-512–1 can be governed by TTYH1 and activates the miR-512–1-LIN28A-MYCN circuit to control the development of embryonal tumors with multilayered rosettes [53]. Interestingly, Anandagoda et al. discovered that the TF FoxP3 bound to the SE region of pri-miR-142 and enhance transcription, suppressing the expression of its downstream target gene PDE3b in regulatory T cells and controlling the tumor inflammatory microenvironment [188].

SE-derived miRNAs mediated by HMEs have been implicated in the development of tumors. SEs and miRNAs are closely correlated for tissue-specific detection, since Oct4, Sox2, Nanog, and mediator-defined SEs and H3K27ac-defined SEs in mESCs mostly overlap; the H3K27ac histone mark is widely employed as a marker to identify SEs [189, 190]. H3K27ac ChIP-seq data were utilized to identify SEs in different tissues and cell lines, and thus, cell-type-specific SE-miRNAs, such as miR-372, were identified [190].

Additionally, the histone lysine demethylase JMJD2A promotes the growth of liver cancer cells via the miR-372-JMJD2AΔ-P21WAF1/Cip1-Pim1-pRB-CDK2-CyclinE-C-myc axis [191]. When linked to the 3'-UTR of the target gene HDAC4, miR-1 functions as a SE-miRNA to prevent the proliferation of lung cancer cells [190, 192]. Specific SE-derived miRNAs exhibit various levels of expression in different cancers and affect diverse tumor characteristics, which may be related to tissue specificity. However, we still do not have a complete grasp of the mechanism underlying these phenomena. SE-associated miRNAs can also be mediated by cofactors in tumors. BRD4 recruitment at the upstream enhancer regions of miR-155 and miR-146a was dramatically increased and activated miR-155 and miR-146a transcription by TNFa stimulation in human umbilical vein endothelial cells [193]. One study showed that SE-miR-155 regulated HuR mRNA levels, migration, and translation in colon cancer cells by targeting AU-rich regions at the 3'-UTR of HuR mRNA [194]. Another study showed that by controlling the IL-17 response in the etiology of colon inflammation and associated cancers, SE-miR-146a was an important negative regulator [195]. Therefore, BET-mediated SE-miRNA miR-155 and miR-146a may significantly impact cancer progression. The creation and alteration of SE-miRNAs during carcinogenesis is linked to genomic instability, gene mutations, and the DNA damage response, which may cause aberrant gene expression and accelerate the malignant development of cancer [170]. Hence, the mechanism by which these SE-miRNAs regulate tumors deserves further study.

### SE-associated circRNAs

CircRNAs are ncRNAs that are covalently closed, expressed in eukaryotes and produced primarily by backsplicing [196]. Due to their lack of PolyA in the 3’ end and capping of the 5’ end, they are resistant to digestion by exonucleases, particularly RNase R, which gives them more stability than linear RNAs [197]. With a longer half-life, circRNAs have become candidates for biomarker studies in human tumors. Most circRNAs function as miRNA sponges; others are considered protein sponges that play roles in tumors. In addition, circRNAs influence alternative splicing via RNA-mediated interactions to regulate the expression of genes and interact with RNA-binding proteins as protein scaffolds, and they are translated into proteins that control life activities. Based on the differences among the sequence types contained within circRNAs, they can be divided into three groups, namely, exonic circRNAs (EcRNA), exon‒intron circRNAs, and intronic circRNAs [198]. EcRNAs account for the majority of circRNAs and are transported to the cytoplasm, while the other two groups containing introns remain in the nucleus. Recently, increasing numbers of SE-circRNAs have been discovered, and they are involved in the malignancy-related functions of tumor cells (Fig. 6).

TFs can regulate SE-associated circRNAs directly in tumor cells. For instance, the TF Meis1 directly binding to the SE at the circNfix locus boosted the production of SE-associated circNfix [199]. CircNFIX knockdown led to the arrest of the cell cycle; inhibition of glycolysis, migration, and invasion; and promotion of apoptosis by regulating the miR-378e/RPN2 axis [199, 200]. Moreover, the proximal regulatory region of SE-associated hsa_circ_0001727 was directly occupied by the TFs USF1 and RXRA in HCC. These results together strongly suggest that SE-associated hsa_circ_0001727 is directly transcriptionally activated by several TFs via its upstream SE region in HCC [201]. Moreover, TFs can also bind to the SE of an intermediate gene to indirectly activate SE-associated circRNAs. For example, QKI, an alternative splicing factor, influences SE-circRNA production of circ-0008150 and circ-0007821, mediated by the YY1/p65/p300 complex in the course of the EMT in the HCC context [86]. More interestingly, some SE-associated circRNAs are activated by HMEs. For example, a substantial SE cluster was found upstream of circ 0001827, which carried a significant number of histone H3 lysine 36 trimethylation (H3K36me3) marks; this histone modification is associated with transcriptional activation and transcriptional repression signals along the gene body, indicating substantial transcriptional elongation in HCC cells [201].

Overall, these findings clearly expand our understanding of circRNA regulatory mechanisms and may have crucial implications for future research on ncRNAs. Although studies on SE-circRNAs and tumors are progressing rapidly, many aspects of SE-circRNAs remain unknown. The mechanisms underlying the production, regulation and biological function of SE-circRNAs remain to be further explored. We believe that SE-circRNAs will be new tools for cancer therapy development in the future.

## Advances in methods used in super-enhancer studies

We summarize available techniques and methods to investigate the identification of SEs and their functions in tumor and regulatory mechanisms (Table 1). First, to identify SEs, bioinformatics databases (dbSUPER, SEA) are required [202, 203]. However, most of the predictions are only theoretically possible and need to be validated with experiments. Therefore, it is essential to identify SEs experimentally. The ChIP-Seq technique combines ChIP with second-generation sequencing technology to identify DNA fragments that interact with HMEs, TF, and other factors [204]. Researchers may collect genome-wide information on DNA segments interacting with TFs, histones, and other proteins by mapping the sequencing data to the genome [205]. Thus, ChIP analysis data of active enhancer locations have revealed that these enhancers are enriched transcriptionally active molecular markers on the genome [206]. Later analysis of all active enhancers led to the discovery of SEs. Subsequently, researchers identified SEs via other methods, such as chromosome conformation capture 3C (chromosome conformation capture) and extended methods, namely, 4C (chromosome conformation capture-on-chip), 5C (chromosome conformation capture carbon copy), and Hi-C (high-throughput chromosome conformation capture technology) [207].

**Table 1:** SE-related databases

Classify	methods	Database	Functions of database	Website	Ref
Identify SEs	ChIP-Seq, 3C, 4C, 5C, Hi-C	dbSUPER	The first comprehensive, interactive database of SEs involved in cellular identity and disease transcriptional control	http://bioinfo.au.tsinghua.edu.cn/dbsuper/	[202]
		SEA	Lncludes SEs from several species and information on how they affect cellular identity	http://sea.edbc.org	[203]
Study the function of SEs	CRISPR/Cas9	Cistrome Cancer	A comprehensive resource for predicted "SE" target genes and enhancer profiles and TF targets	http://cistrome.org/CistromeCancer/	[208]
		SEA version 3.0	A comprehensive database covering all available SE information for multiple species and will facilitate the study of SE function	http://sea.edbc.org	[209]
Regulatory mechanisms of SEs	3C, 4C, 5C, Hi-C, ChIP-seq,ATAC-Seq, CUT&Tag, CRISPR/Cas9	SE analysis	A database used to analyze the regulatory mechanisms of SEs	http://licpathway.net/SEanalysis	[67]
		SEdb	A database that provides basic information about SEs and can also combine, evaluate, and disclose the regulatory mechanisms of SEs	http://www.licpathway.net/sedb	[210]
		SELER	SE-lncRNA transcriptional regulation in human malignancies	http://www.seler.cn	[211]
		TRCirc	Mainly includes transcription of circRNAs and partial SE-circRNAs	http://www.licpathway.net/TRCirc	[212]

More interestingly, CRISPR/Cas9 enables precise editing of specific sites in the genome [213]. It is based on the principle that endonuclease the Cas9 protein recognizes specific genomic sites and cuts double-stranded DNA by guiding RNA and double-stranded DNA, and cell machinery repairs the DNA via non-homologous end joining or homologous recombination to achieve DNA level knockout or precise editing. The function of these SEs may be examined using sophisticated CRISPR/Cas9 genome editing tools to discover their associated regulatory elements in various ways. Furthermore, editing the target SEs with CRISPR/Cas9 technology may be used to investigate and validate the role of SEs in cancer [214]. Similarly, the SE-related databases (Cistrome Cancer, SEA version 3.0, etc.) are based on CRISPR/Cas9 technology and can be used to research the functions of SEs [208, 209].

Moreover, more research must be done on the regulatory mechanisms of SEs. With the improvement in bioinformatics algorithms and high-throughput sequencing technologies, the interaction among chromatin sequences can be directly analyzed by 3C, 4C, 5C, or Hi-C technology, and the genes associated with SEs can be identified. Based on these techniques, several SE databases (SEdb, SE analysis) have been developed to annotate the potential functions of SEs involved in gene regulation [66, 210]. In addition, using CRISPR gene-editing technology, single-cell sequencing technology combined with ChIP-seq, ATAC-Seq, and CUT&Tag can be used to study the transcriptional regulatory elements of SEs in depth [215]. Interestingly, several SE databases (SELER, TRCirc) have been developed and can be used to analyze the transcriptional regulatory mechanisms of SE-ncRNAs [211, 212]. Through these datasets, we may gain a better understanding of SE regulatory mechanisms and thus establish a solid theoretical foundation for cancer medication development.

## SE and SE complexes function as potential biomarkers in cancer

Applying epigenetics for early cancer detection is becoming a popular approach, and SEs and SE-complexes have also received attention for use in cancer prognosis. SEs mediated by SE complexes are sets of transcription enhancers critical for maintaining cellular properties and driving the expression of genes unique to a particular cell type. During tumorigenesis, cancer cells produce SEs of oncogenes and other genes. However, normal cells do not express critical oncogenes regulated via SEs produced in cancer cells, which indicates that SEs maintain tumor characteristics by regulating these essential genes and play vital roles in the occurrence and development of cancer. Functional SEs can be used as transcriptional units to recruit RNA pol II and generate eRNAs via transcription, which has special implications for the clinical application of SEs. The expression level of an eRNA is positively correlated with the activity of its SE, which may cooperate with other SEs to activate gene transcription, suggesting the that detection of eRNA is a new method to identify SEs. eRNA expression can be measured by RT‒qPCR at relatively low cost and through easy application in the clinic. Therefore, eRNAs are expected to become new markers for tumor screening and precision diagnosis and treatment in the future.

Finding these complexes will aid in diagnosing cancers since SE complexes play a regulatory function in the crucial pathways involved in tumor growth. Therefore, SE complexes can be potentially employed as biomarkers for tumor diagnosis. As a reliable diagnostic indicator of BPDCN, the SE-complex TCF4 was discovered by Ceribelli et al., and its downregulation results in loss of the BPDCN-specific gene expression program and apoptosis [82]. Previous studies have revealed that SE complexes are associated with tumor prognosis. For instance, human HCCs exhibit much higher levels of overexpressed SE complex elements such as MED1, EP300, BRD4, and CDK7, which are highly associated with a poor prognosis for HCC patients [120]. Moreover, Yang et al. discovered that the expression of C/EBP, which is a component of a SE complex, is highly correlated with tumor stage and a shorter survival time, making it a potential prognostic marker for HCC [216]. These studies show that SE complexes can be used as diagnostic markers of tumors and are useful for the diagnosis of early and advanced tumors. They also suggest new possibilities for the prognostic evaluation of tumor patients.

More interestingly, SE-associated genes can offer some guidance for cancer diagnosis. For example, researchers discovered that liver-specific SE-driven lncRNA-DAW was a useful clinical diagnostic biomarker for HCC. In addition, Baldauf et al. provided evidence that the SE-driven EWSR1-ETS targets BCL11B and GLG1 and can be identified using immunohistochemistry to quickly and accurately diagnose Ewing sarcoma [217]. Moreover, SE-related genes can play a guiding role in cancer prognosis. Xing et al. revealed that some structural rearrangement patterns are evident in the SE region of the ZFP36L2 gene expressed in gastric cancers, and they may be associated with poor prognoses [218]. Furthermore, the SE-related genes MANBAL, KCMF1, and AHCY were highly expressed in oral squamous cell carcinoma (OSCC) induced by TFDP1 and were closely associated with the prognosis of OSCC [219]. These findings indicate that SE-associated genes might be used as specific diagnostic or prognostic markers of tumors.

## Clinical application of super-enhancer small-molecule inhibitors

An increasing body of evidence suggests that SEs play a crucial role in tumor growth and that oncogenic SEs induce oncogene transcription and continually increase cell survival and proliferation, implying that inhibiting SEs is a viable anticancer treatment [215]. However, it is challenging to use SEs as direct targets for tumor therapy due to the binding of multiple regulatory elements to SEs and the lengths of SEs. Oncogene transcription is facilitated by SE complexes, which function as structural cores of transcription complexes. As a result, targeting a SE complex and inhibiting oncogene transcription has gained popularity as a cancer treatment method [132]. The same oncogene can create a diverse SE landscape in various tumor forms but is unique to cancer cells. Since various tumor cells share common SE components, we might be able to inhibit these components directly and prevent an oncogene from developing an addiction to SEs. According to reports, the interactions of the SE complex are necessary for SE activity. Therefore, the design of targeted SE-complex inhibitors in tumor cells is considered a promising approach. Furthermore, SE-complex inhibitors might disrupt the interaction between the SE in one area and its associated complexes, inhibiting oncogenes expression. In summary, inhibitors targeting the SE complex have become a new therapeutic option. Currently, the main therapeutic drugs for cancers targeting SE complex inhibitors include TF, HME, and SE cofactor inhibitors [220] (Table 2).

**Table 2:** Small-molecule inhibitors targeting SE

Target		Small-molecule inhibitor	Disease	Status	Phase	NCT number
TF	KLF5	SR18662	Colorectal Cancer [221]	–-	–-	–-
		ML264	Colorectal Cancer [221]	–-	–-	–-
			Breast Cancer [222]	–-	–-	–-
	TCF4	LF3	Colon Cancer [223]	–-	–-	–-
	MYC	MYCi361	Prostate Cancer [224]	–-	–-	–-
		MYCi975	Glioma [117]	–-	–-	–-
		EN4	Breast Cancer [225]	–-	–-	–-
HMEs	HDAC3	RGFP966	Hepatocellular Carcinoma [226]	–-	–-	–-
	HDAC1, HDAC2, HDAC3	Domatinostat (4SC-202)	Merkel Cell Carcinoma [227]	–-	–-	–-
			Advanced Hematologic Malignancies	Completed	1	NCT01344707
		Tucidinostat	Non Small Cell Lung Cancer	Recruiting	2	NCT05141357
			Cervical Cancer	Recruiting	1/2	NCT04651127
		PCI-24781	Lymphoma Follicular	Recruiting	2	NCT03934567
			Metastatic Solid Tumors	Recruiting	1	NCT01543763
			Sarcoma	Completed	1/2	NCT01027910
		Largazole	Colorectal Cancer [228]	–-	–-	–-
	HDAC1, HDAC3, HDAC7	MS-275 (Entinostat)	Melanoma	Recruiting	2	NCT03765229
			Breast Cancer	Recruiting	1	NCT03473639
		MGCD0103	Rhabdomyosarcoma	Recruiting	1	NCT04299113
			Urothelial Carcinoma	Completed	2	NCT02236195
		Vorinostat	Non-small Cell Lung Cancer	Terminated	1/2	NCT00503971
			Lymphoma	Completed	3	NCT01728805
			Multiple Myeloma	Completed	1/2	NCT01502085
		Panobinostat	Mesothelioma	Completed	1	NCT00535951
			Non Small Cell Lung Cancer	Completed	1	NCT00535951
	JMJD2A	Z-JIB-04	Breast Cancer [229]	–-	–-	–-
	KDM6A	GSK J1	Glioblastoma [230]	–-	–-	–-
			Neck Squamous Cell Carcinoma [231]	–-	–-	–-
	EP300	CPI-637	Prostate Cancer [232]	–-	–-	–-
		Y08197	Prostate Cancer [232]	–-	–-	–-
Cofactors	BRD4	AZD5153	Malignant Solid Tumor	Completed	1	NCT03205176
			Lymphadenoma	Completed	1	NCT03205176
		ABBV-075	Prostate Cancer	Completed	1	NCT02391480
			Breast Cancer	Completed	1	NCT02391480
			Multiple Myeloma	Completed	1	NCT02391480
		BMS-986158	Advanced Cancer	Completed	1/2	NCT02419417
			Solid Tumor Childhood	Recruiting	1	NCT03936465
			Lymphoma	Recruiting	1	NCT03936465
			Brain Tumor	Recruiting	1	NCT03936465
		CPI-0610	Myelofifibrosis	Recruiting	2	NCT02158858
		PLX51107	Sarcoma	Terminated	2	NCT02683395
			Breast Cancer	Terminated	2	NCT02683395
			Uveal Melanoma	Terminated	2	NCT02683395
		INCB054329	Ovarian Cancer	Terminated	1/2	NCT02431260
			Lymphoma	Terminated	1/2	NCT02431260
		FT-1101	Acute myeloid leukemia	Completed	1	NCT02543879
		iBET151	Gastric cancer [233]	–-	–-	–-
		JQ1	Prostate Cancer [234]	–-	–-	–-
			Ovarian Cancer [235]	–-	–-	–-
			Multiple Myeloma Cells [236]	–-	–-	–-
	BRD2, BRD4	CC-90010	Glioblastoma	Recruiting	1	NCT04324840
			Astrocytoma	Active not recruiting	1	NCT04047303
	BRD2, BRD3, BRD4	GSK525762	NUT Carcinoma	–-	1	NCT01597703
		OTX-015	Acute Leukemia	Completed	1	NCT01713582
			Lymphoma	Completed	1	NCT01713582
			NUT Midline Carcinoma	Completed	1	NCT02259114
			Adenocarcinoma	Completed	1	NCT02259114
			Triple Negative Breast Cancer	Completed	1	NCT02259114
	BRDT, BRD2, BRD3, BRD4	ODM-207	CRPC	–-	1	NCT03035591
			Melanoma	–-	1	NCT03035591
			Non-Medullary Carcinoma	–-	1	NCT03035591
Cofactors	CDK7	SY-1365	Ovarian Cancer	Recruiting	1	NCT03134638
			Breast Cancer	Recruiting	1	NCT03134638
		CT7001	Advanced Solid Malignancies	Recruiting	2	NCT03363893
		THZ1	Neuroblastoma [40]	–-	–-	–-
			Glioma [237]	–-	–-	–-
	CDK9	Dinaciclib	Hepatocellular carcinoma [238]	–-	–-	–-
			Acute Myeloid Leukemia	Active not recruiting	1	NCT03484520
			Malignant Solid Neoplasm	Recruiting	1	NCT01434316
		Wogonin	Lung Epithelial Cancer [239]	–-	–-	–-
			Hepatocellular Carcinoma [240]	–-	–-	–-
		BAY 1143572	Leukemia	Completed	1	NCT02345382
			Neoplasms	Completed	1	NCT01938638
	CDK4, CDK6	Trilaciclib	Small cell lung cancer	Completed	1/2	NCT02499770
		PD-0332991	Lung cancer	Recruiting	1	NCT03170206
			Metastatic Pancreatic Ductal Adenocarcinoma	Completed	1	NCT02501902
		Ribociclib (LEE011)	Non-small cell lung cancer	Completed	1	NCT02292550
			Breast Cancer	Recruiting	4	NCT04657679
			Ovarian Carcinoma	Recruiting	1	NCT04315233
		SPH3643	Colon Cancer [241]	–-	–-	–-
			Glioblastoma [241]	–-	–-	–-
	CDK12, CDK13	THZ531	Prostate Cancer [242]	–-	–-	–-
	CDK8, CDK19	BCD-115	Breast Cancer	Completed	1	NCT03065010

### TF inhibitors

Directly targeting SE complex components such as TF KLF5, TCF4, and MYC are valuable chemical probes and potential anticancer drugs. A small-molecule drug called ML264, which blocks the SE-complex KLF5, slows the development of colorectal cancer both in vivo and in vitro, encouraging breast cancer cell growth [221, 222]. A particular Wnt signal inhibitor with anticancer action is called LF3, and it plays an inhibitory role by destroying the interaction between β-catenin and the SE-complex TCF4; it reduced tumor growth and induced cell differentiation in a colon cancer xenotransplantation model [223]. In addition, MYCi361, a small-molecule Myc inhibitor, significantly inhibited PCa cell proliferation [224]. Myci975, another small-molecule Myc inhibitor, inhibited TMEM44-AS1-induced glioma cell growth [116]. In summary, SE complex-related TF inhibitors significantly reduce tumor growth and tumor cell proliferation.

### HMEs inhibitors

HDAC is an enzyme that removes acetyl groups from lysine residues on histone proteins and thus alter chromosome shape and control gene expression [243]. Because it can increase the activity of TFs and control the expression of tumor suppressor genes, it has become the latest and most popular target in tumor chemotherapy drug research. Selective and broad-spectrum histone deacetylase inhibitors (HDACIs) disrupt SEs globally to influence carcinogenesis [244]. The HDACI largazole reduces oncogenes that promote colorectal cancer cell transformation by selectively targeting SEs in transformed cells [228]. HDAC inhibitors (panobinostat, vorinostat, romidepsin) induce metabolic reprogramming by targeting SEs in glioblastoma [244]. A novel and versatile HDAC inhibitor called panostat (LBH589, NVP-LBH589) stops the development of mesothelioma and small-cell lung cancer cells [245]. Furthermore, histone acetylating transferases CBP/p300 and histone methyltransferases MLL3/MLL4 are major SE epigenomic writers. The CBP/EP300 inhibitors Y08197 and CPI-637 inhibited PCa cell migration, suggesting that Y08197 and CPI-637 have potential inhibitory effects on PCa cell metastasis [232]. These studies suggest that HME inhibitors can play an essential role in cancer therapy by targeting SE complexes.

### SE cofactor inhibitors

SE cofactor inhibitors mainly include BRD family protein inhibitors and CDK inhibitors. They inhibit SE cofactors and regulate SEs, thereby affecting tumor progression. The four members of the BRD family (BRDT, BRD2, BRD3, and BRD4) might be viable targets for developing small-molecule medicines [246]. BET inhibitors specifically disrupt the binding activity of BRD4, one of the SE complex components, decreasing the production of SE-driven oncogenes and attenuating cancer cell proliferation. JQ1 was the first BET inhibitor to target the BET bromodomain. JQ1 binds to the brominated domains of all four BET family members, thereby preventing BRD4 from interacting with acetylated-modified proteins. This limits BRD4 binding to the H3K27ac site and SEs and inhibits the ability of SEs to interact with promoters, decreasing oncogene transcription [247]. Treatment of myeloma cells with JQ1 resulted in the selective deletion of BRD4 on SEs and revealed the presence of SE-related genes in MM cells [236]. JQ1 has been reported to deactivate inhibitory aldehyde dehydrogenase activity in ovarian cancer cells by inhibiting eRNA in BRD4-regulated super enhancers [235]. SE-related genes have been discovered to be downregulated in AML by I-BET151, a different BET inhibitor that targets BRD4 [29]. Another BET inhibitor that efficiently inhibits cell proliferation in vivo and in vivo in a range of hematologic malignancies and solid tumors is OTX015, which targets BRD2, BRD3, and BRD4 [248].

Various tumor-associated SEs have been targeted by CDK small-molecule inhibitors. Tumor cells mediated by SEs are susceptible to THZ1, a specific CDK7 inhibitor. THZ1 covalently binds to the cysteine at position 312 of CDK7 to limit the kinase activity of CDK7, inhibiting the serine phosphorylation of Pol II CTD at position 5, thereby inhibiting transcription initiation and further preventing Pol II from pausing proximal to the promoter. After THZ1 treatment, the SE activity decreases, leading to the transcriptional repression of multiple oncogenes, thereby inhibiting the growth and proliferation of various tumor cells [249]. For example, in MYCN-amplified neuroblastoma cells, THZ1 was discovered to preferentially disrupt the global transcriptional regulation of MYCN [39]. Additionally, THZ1 specifically inhibited oncogenic SE activity in ESCC cells [132]. A recent study showed that patients with glioma had a poor prognosis when CDK7 expression was high. However, the transcription of almost all the genes in glioma cells was significantly altered after THZ1 treatment, with SE-related genes being the most markedly affected [237]. Interestingly, SY-1365, a different Syros-developed targeted CDK7 inhibitor, markedly suppresses several solid cancers, including blood malignancies and breast cancer, ovarian cancer, small-cell lung cancer, AML, and acute lymphoblastic leukemia [250].

## Further discussion and review

Basic theories of SE biology and its function have been reported over the past decades. Extensive studies have revealed that SEs promote the overexpression of oncogenes, and specific cancer treatment pathways may include altering SE structures and complexes. However, what we know about SEs is only the tip of the iceberg. What is an appropriate meaning of the word "SE"? Technologically, the methods used to identify SEs need to be improved. ROSE and other currently employed methods can fail to locate a true SE. Since SEs are operationally not functionally defined, we cannot distinguish between true enhancers that are not canonically labeled. Moreover, ROSE has shortcomings based on the need to consider the spatial configurations of SEs and is somewhat limited in the recognition of SE-driven ncRNAs. Moreover, it is crucial to determine whether SEs have any observable characteristics that set them apart from TFs.

Recent research indicates that the component enhancers constituting a SE undergo various contributing interactions, including temporal linkages and absolute, synergistic, interdependent, hierarchical, and redundant interactions. Despite the many aforementioned SE functions, recent findings imply that only a few individual enhancers, even in the densest enhancer clusters, are critical to target gene activity. Furthermore, a single enhancer is equally likely as a SE to control highly expressed genes. Does the integration of diverse component enhancers in a given SE function in various environments or in a spatiotemporal manner? Is the activity of each enhancer that makes up a SE in cancer cells consistent with the corresponding enhancer activity in normal cells? How does the altered activity of a signaling pathway affect the assembly of individual enhancers in SEs? Moreover, we may acquire novel ideas about the function of SEs in the future. Because numerous articles have revealed SEs in intranuclear genetic material, we hypothesize that SEs in mitochondrial genetic material may be crucial for carcinogenesis and cell identity.

Although the PS of TFs at SEs suggests an intriguing model of SE function in regulating numerous spatially proximal genes, many crucial questions still need to be answered. For example, what triggers the production of phase-separated condensates at SEs? Future modeling and research on the functions of biomolecular polymers such as DNA, RNA, and proteins in condensate behaviors may benefit from the establishment of related physical and chemical principles. Recently, the molecular mechanisms underlying the de novo acquisition of cancer-specific SEs and CRCs in various cancer types were described. However, whether and how SEs and CRCs affect medication resistance and relapse is uncertain, and further research is necessary. Additionally, investigations into the pharmacological targets of the proteins that comprise CRCs and how they interact with eRNAs are crucial.

Increasing evidence shows that SEs are essential for regulating the expression of oncogenes in tumors. The relationships between SE and targeting oncogenes may be based on one-to-many and many-to-one coupling, which leads to the following problem: how can a targeted gene be accurately regulated by SEs? SEs function as oncogenes in most cancers to promote tumor development. Recent studies have also suggested that SEs drive tumor suppressor genes to participate in the development of tumors. For example, *Mll4* deletion downregulates tumor suppressor genes (e.g., Bcl6 and Dnmt3a) by generally decreasing the abundance of SEs and H3K4me3 and causing widespread degradation of these epigenomic marks during medulloblastoma generation [30]. The tumor suppressors ETV6, CEBPA, IRF8, and IRF1 are among the lineage-controlling master TFs found in CD141-positive monocytes enriched with SE-associated genes upregulated by cortistatin A [29]. Interestingly, the SE-driven lncRNA RP11-569A11.1 reduced cell growth and metastasis by modulating IFIT2 in colorectal cancer [251]. These findings suggest a novel tumor-suppressive mechanism and support a function for SEs in the prevention of tumors. However, how SEs that suppress tumors are specifically generated or whether they aid in tumor suppression in normal cells is unknown.

All the components of a SE complex play vital roles in the treatment of disease. In the field of cancer therapy, inhibitors of SE complex proteins, such as BRD4, CDK7, or CDK9, show great potential. Nevertheless, the fundamental mechanism underlying the exceptional sensitivity of SE-associated genes to the inhibitors of SE components remains unknown. The excessive loading of BRD4 and mediators at SE sites linked to MYC and other lineage-specific survival genes may be preferentially replaced by JQ1, which would explain why JQ1 is insensitive to MM treatment [114]. THZ1 can also cause abnormalities in cotranscriptional capping, elongation, promoter proximal pausing, and Pol II phosphorylation in addition to inhibiting SEs. However, since practically all human tissues include "normal" SEs that indicate tissue specificity, the possibility that these inhibitors might disrupt fundamental biological processes with eventual global effects on gene expression cannot be disregarded. Unanswered issues include the contribution of various components to the different activities of SEs as they pertain to cancer and how they preferentially respond to inhibitory treatment. Before identifying further targetable nodes, the roles of each element in a SE complex and how inhibition affects it must be explained in detail.

Although SE inhibitors are currently used in some malignancies, including PCa, lymphoma, and breast cancer, the understanding of the biology of the different members of the SE complexes is limited. Notably, targeting SEs by inhibitors of SE complexes as a cancer treatment may result in notable adverse effects since the expression of several cancer-suppressing genes would also be reduced, which may explain why JQ1 cannot produce intense antitumor activity in solid tumors. Hence, further research and a deeper comprehension of the processes by which SEs promote tumor suppression are required before SEs can be utilized as therapeutic targets in cancer. Intensive investigation into the relationship between transcriptional addiction and cancer will benefit the development of more-relevant inhibitors. For instance, CDK9 inhibition is an efficient therapeutic approach for CLL [252]. However, to date, the medicines created have only exerted a limited therapeutic effect, and there are no indicators of pharmacological success. Finding remedies via synergistic therapeutics may be possible when the processes that cause these dependencies are elucidated.

## Data Availability

Not applicable.

## Abbreviations

ACC: Adenoid cystic cancer
AML: Acute myeloid leukemia
AR: Androgen Receptor
ATLL: Adult T-cell leukemia/lymphoma
BET: Bromodomain and extraterminal domain
BPDCN: Blastic plasmacytoid dendritic cell neoplasm
BRDs: Bromodomain proteins
CDK: Cyclin-dependent kinase
CeRNAs: Competing endogenous RNAs
ChIP: Chromatin immunoprecipitation
ChIP-seq: Chromatin immunoprecipitation sequencing
CircRNAs: Circular RNAs
CLL: Chronic lymphocytic leukemia
CR: Core regulatory
CRC: Core regulatory circuit
CRPC: Castration-resistant prostate cancer
CSCs: Cancer Stem Cells
CTD: Carboxy-terminal domain
3C: Chromosome conformation capture
4C: Chromosome conformation capture-on-chip
5C: Chromosome conformation capture carbon copy
3D: Three-dimensional
EAC: Esophageal adenocarcinoma
EBV: Epstein-Barr virus
EcRNA: Exonic circRNAs
EMT: Epithelial-mesenchymal transition
EnCRISPRa: Enhancer-targeting CRISPR epigenetic editing activation system
ERBSs: Estrogen receptor alpha binding sites
ERNAs: Enhancer RNAs
ESC: Embryonic stem cell
ESCC: Esophageal squamous cell carcinoma
HCC: Hepatocellular carcinoma
HDAC: Histone deacetylase
HDACIs: Histone deacetylase inhibitors
Hi-C: High-throughput chromosome conformation capture technology
HMEs: Histone-modifying enzymes
HNSCC: Head and Neck Squamous Cell Carcinoma
HPV: Human papillomavirus
HTLV-1: Human T-cell lymphotropic virus type 1
H3K27ac: Histone H3 lysine 27 acetylation
H3K4me1: Histone H3 lysine 4 mono-methylation
H3K79me2: Histone H3 lysine 79 dimethylation
H3K36me3: Histone H3 lysine 36 trimethylation
LASP1: LIM and SH3 domain protein 1
LncRNA: Long noncoding RNA
LUAD: Lung adenocarcinoma
MiRNAs: MicroRNAs
MM: Multiple Myeloma
NcRNA: Noncoding RNA
NFR: Nucleosome-free region
NuRD: Nucleosome-remodeling deacetylase
OSCC: Oral squamous cell carcinoma
PCa: Prostate cancer
PD-L1: Programmed death-ligand 1
PS: Phase Separation
P-TEFb: Positive transcription elongation factor-b
SCCs: Squamous cell carcinomas
SE: Super-enhancer
SE-miRNAs: Super-enhancer-associated miRNAs
SNP: Single-nucleotide polymorphism
TAD: Topologically associating domain
T-ALL: T-cell acute lymphoblastic leukemia
TE: Typical enhancers
TF: Transcription factors
TSS: Transcription start site

## References

[1] Cao H, Zhuo R, Zhang Z, Wang J, Tao Y, Yang R (2022). Super-enhancer-associated INSM2 regulates lipid metabolism by modulating mTOR signaling pathway in neuroblastoma. Cell Biosci.

[2] Whyte WA, Orlando DA, Hnisz D, Abraham BJ, Lin CY, Kagey MH (2013). Master transcription factors and mediator establish super-enhancers at key cell identity genes. Cell.

[3] Lee T, Young R (2013). Transcriptional regulation and its misregulation in disease. Cell.

[4] Calo E, Wysocka J (2013). Modification of enhancer chromatin: what, how, and why?. Mol Cell.

[5] Liu Q, Guo L, Lou Z, Xiang X, Shao J (2022). Super-enhancers and novel therapeutic targets in colorectal cancer. Cell Death Dis.

[6] Belloucif Y, Lobry C. Super-Enhancers Dysregulations in Hematological Malignancies. Cells. 2022;11(2):196. 10.3390/cells11020196 PMC8774084 35053311

[7] Boland M, Nazor K, Loring J (2014). Epigenetic regulation of pluripotency and differentiation. Circ Res.

[8] Tang F, Yang Z, Tan Y, Li Y (2020). Super-enhancer function and its application in cancer targeted therapy. NPJ Precis Oncol.

[9] Mueller D, Bach C, Zeisig D, Garcia-Cuellar M, Monroe S, Sreekumar A (2007). A role for the MLL fusion partner ENL in transcriptional elongation and chromatin modification. Blood.

[10] Kagey M, Newman J, Bilodeau S, Zhan Y, Orlando D, van Berkum N (2010). Mediator and cohesin connect gene expression and chromatin architecture. Nature.

[11] Hnisz D, Abraham BJ, Tong IL, Lau A, Saintandré V, Sigova AA, et al. Transcriptional super-enhancers connected to cell identity and disease. Cell. 2013;155(4):934–47. 10.1016/j.cell.2013.09.053 PMC3841062 24119843

[12] Huang J, Li K, Cai W, Liu X, Zhang Y, Orkin SH (2018). Dissecting super-enhancer hierarchy based on chromatin interactions. Nat Commun.

[13] Chen Y, Yao B, Zhu Z, Yi Y, Lin X, Zhang Z (2004). A constitutive super-enhancer: homologous region 3 of Bombyx mori nucleopolyhedrovirus. Biochem Biophys Res Commun.

[14] Li Y, Rivera C, Ishii H, Jin F, Selvaraj S, Lee A (2014). CRISPR reveals a distal super-enhancer required for Sox2 expression in mouse embryonic stem cells. PLoS One.

[15] Novo CL, Javierre BM, Cairns J, Segonds-Pichon A, Wingett SW, Freire-Pritchett P (2018). Long-Range Enhancer Interactions Are Prevalent in Mouse Embryonic Stem Cells and Are Reorganized upon Pluripotent State Transition. Cell Rep.

[16] Li K, Liu Y, Cao H, Zhang Y, Gu Z, Liu X (2020). Interrogation of enhancer function by enhancer-targeting CRISPR epigenetic editing. Nat Commun.

[17] Wang X, Cairns MJ, Yan J (2019). Super-enhancers in transcriptional regulation and genome organization. Nucleic Acids Res.

[18] Niederriter AR, Varshney A, Parker SC, Martin DM (2015). Super Enhancers in Cancers, Complex Disease, and Developmental Disorders. Genes (Basel).

[19] Jia Q, Chen S, Tan Y, Li Y, Tang F (2020). Oncogenic super-enhancer formation in tumorigenesis and its molecular mechanisms. Exp Mol Med.

[20] D'Alessio AC, Fan ZP, Wert KJ, Baranov P, Cohen MA, Saini JS (2015). A Systematic Approach to Identify Candidate Transcription Factors that Control Cell Identity. Stem Cell Rep.

[21] Ryu J, Kim H, Yang D, Lee AJ, Jung I (2019). A new class of constitutively active super-enhancers is associated with fast recovery of 3D chromatin loops. BMC Bioinformatics.

[22] Grosveld F, van Staalduinen J, Stadhouders R (2021). Transcriptional Regulation by (Super)Enhancers: From Discovery to Mechanisms. Annu Rev Genomics Hum Genet.

[23] Wang J, Lin B, Zhang Y, Ni L, Hu L, Yang J (2020). The Regulatory Role of Histone Modification on Gene Expression in the Early Stage of Myocardial Infarction. Front Cardiovasc Med.

[24] Schoenfelder S, Fraser P (2019). Long-range enhancer-promoter contacts in gene expression control. Nat Rev Genet.

[25] Furlong E, Levine M (2018). Developmental enhancers and chromosome topology. Science (New York, NY).

[26] Wang Q, Sun Q, Czajkowsky D, Shao Z (2018). Sub-kb Hi-C in D. melanogaster reveals conserved characteristics of TADs between insect and mammalian cells. Nat Commun..

[27] Deng R, Huang JH, Wang Y, Zhou LH, Wang ZF, Hu BX (2020). Disruption of super-enhancer-driven tumor suppressor gene RCAN1.4 expression promotes the malignancy of breast carcinoma. Mol Cancer.

[28] Li X, Zhou B, Chen L, Gou LT, Li H, Fu XD (2017). GRID-seq reveals the global RNA-chromatin interactome. Nat Biotechnol.

[29] Pelish HE, Liau BB, Nitulescu II, Tangpeerachaikul A, Poss ZC, Da Silva DH (2015). Mediator kinase inhibition further activates super-enhancer-associated genes in AML. Nature.

[30] Dhar SS, Zhao D, Lin T, Gu B, Pal K, Wu SJ (2018). MLL4 Is Required to Maintain Broad H3K4me3 Peaks and Super-Enhancers at Tumor Suppressor Genes. Mol Cell.

[31] Kumar V, Westra H, Karjalainen J, Zhernakova D, Esko T, Hrdlickova B (2013). Human disease-associated genetic variation impacts large intergenic non-coding RNA expression. PLoS Genet.

[32] Hnisz D, Schuijers J, Lin CY, Weintraub AS, Abraham BJ, Lee TI (2015). Convergence of developmental and oncogenic signaling pathways at transcriptional super-enhancers. Mol Cell.

[33] Mansour M, Abraham B, Anders L, Berezovskaya A, Gutierrez A, Durbin A (2014). Oncogene regulation. An oncogenic super-enhancer formed through somatic mutation of a noncoding intergenic element. Science (New York, NY).

[34] Oldridge DA, Wood AC, Weichert-Leahey N, Crimmins I, Sussman R, Winter C (2015). Genetic predisposition to neuroblastoma mediated by a LMO1 super-enhancer polymorphism. Nature.

[35] Kandaswamy R, Sava GP, Speedy HE, Beà S, Martín-Subero JI, Studd JB (2016). Genetic Predisposition to Chronic Lymphocytic Leukemia Is Mediated by a BMF Super-Enhancer Polymorphism. Cell Rep.

[36] Zhang X, Choi PS, Francis JM, Imielinski M, Watanabe H, Cherniack AD (2016). Identification of focally amplified lineage-specific super-enhancers in human epithelial cancers. Nat Genet.

[37] Herranz D, Ambesi-Impiombato A, Palomero T, Schnell S, Belver L, Wendorff A (2014). A NOTCH1-driven MYC enhancer promotes T cell development, transformation and acute lymphoblastic leukemia. Nat Med.

[38] Shi J, Whyte W, Zepeda-Mendoza C, Milazzo J, Shen C, Roe J (2013). Role of SWI/SNF in acute leukemia maintenance and enhancer-mediated Myc regulation. Genes Dev.

[39] Chipumuro E, Marco E, Christensen CL, Kwiatkowski N, Zhang T, Hatheway CM (2014). CDK7 inhibition suppresses super-enhancer-linked oncogenic transcription in MYCN-driven cancer. Cell.

[40] Gröschel S, Sanders M, Hoogenboezem R, de Wit E, Bouwman B, Erpelinck C (2014). A single oncogenic enhancer rearrangement causes concomitant EVI1 and GATA2 deregulation in leukemia. Cell.

[41] Drier Y, Cotton M, Williamson K, Gillespie S, Ryan R, Kluk M (2016). An oncogenic MYB feedback loop drives alternate cell fates in adenoid cystic carcinoma. Nat Genet.

[42] Northcott P, Lee C, Zichner T, Stütz A, Erkek S, Kawauchi D (2014). Enhancer hijacking activates GFI1 family oncogenes in medulloblastoma. Nature.

[43] Kloetgen A, Thandapani P, Tsirigos A, Aifantis I (2019). 3D Chromosomal Landscapes in Hematopoiesis and Immunity. Trends Immunol.

[44] Weischenfeldt J, Dubash T, Drainas A, Mardin B, Chen Y, Stütz A (2017). Pan-cancer analysis of somatic copy-number alterations implicates IRS4 and IGF2 in enhancer hijacking. Nat Genet.

[45] Evans S, Horrell J, Neretti N (2019). The three-dimensional organization of the genome in cellular senescence and age-associated diseases. Semin Cell Dev Biol.

[46] Zhou H, Schmidt S, Jiang S, Willox B, Bernhardt K, Liang J (2015). Epstein-Barr virus oncoprotein super-enhancers control B cell growth. Cell Host Microbe.

[47] Liang J, Zhou H, Gerdt C, Tan M, Colson T, Kaye KM (2016). Epstein-Barr virus super-enhancer eRNAs are essential for MYC oncogene expression and lymphoblast proliferation. Proc Natl Acad Sci U S A.

[48] Chen X, Loo J, Shi X, Xiong W, Guo Y, Ke H (2018). EGFRE6 Protein Expressed by High-Risk HPV Activates Super-Enhancers of the and Oncogenes by Destabilizing the Histone Demethylase KDM5C. Can Res.

[49] Nakagawa M, Shaffer AL, Ceribelli M, Zhang M, Wright G, Huang DW (2018). Targeting the HTLV-I-Regulated BATF3/IRF4 Transcriptional Network in Adult T Cell Leukemia/Lymphoma. Cancer Cell.

[50] Long M, Betrán E, Thornton K, Wang W (2003). The origin of new genes: glimpses from the young and old. Nat Rev Genet.

[51] Siepel A (2009). Darwinian alchemy: Human genes from noncoding DNA. Genome Res.

[52] Benbarche S, Lopez CK, Salataj E, Aid Z, Thirant C, Laiguillon MC (2022). Screening of ETO2-GLIS2-induced Super Enhancers identifies targetable cooperative dependencies in acute megakaryoblastic leukemia. Sci Adv.

[53] Sin-Chan P, Mumal I, Suwal T, Ho B, Fan X, Singh I (2019). A C19MC-LIN28A-MYCN Oncogenic Circuit Driven by Hijacked Super-enhancers Is a Distinct Therapeutic Vulnerability in ETMRs: A Lethal Brain Tumor. Cancer Cell.

[54] Babu D, Fullwood M (2017). Expanding the effects of ERG on chromatin landscapes and dysregulated transcription in prostate cancer. Nat Genet.

[55] Darling A, Shorter J (2021). Combating deleterious phase transitions in neurodegenerative disease. Biochim Biophys Acta.

[56] Brangwynne C, Eckmann C, Courson D, Rybarska A, Hoege C, Gharakhani J (2009). Germline P granules are liquid droplets that localize by controlled dissolution/condensation. Science (New York, NY).

[57] Boeynaems S, Alberti S, Fawzi N, Mittag T, Polymenidou M, Rousseau F (2018). Protein Phase Separation: A New Phase in Cell Biology. Trends Cell Biol.

[58] King J, Shakya A (2021). Phase separation of DNA: From past to present. Biophys J.

[59] Strom A, Emelyanov A, Mir M, Fyodorov D, Darzacq X, Karpen G (2017). Phase separation drives heterochromatin domain formation. Nature.

[60] Cho W, Spille J, Hecht M, Lee C, Li C, Grube V (2018). Mediator and RNA polymerase II clusters associate in transcription-dependent condensates. Science (New York, NY).

[61] Gurumurthy A, Shen Y, Gunn EM, Bungert J (2019). Phase Separation and Transcription Regulation: Are Super-Enhancers and Locus Control Regions Primary Sites of Transcription Complex Assembly?. BioEssays.

[62] Wang C, Lu H, Liu X, Gao X, Tian W, Chen H (2022). A natural product targets BRD4 to inhibit phase separation and gene transcription. iScience.

[63] Tomazou EM, Sheffield NC, Schmidl C, Schuster M, Schönegger A, Datlinger P (2015). Epigenome mapping reveals distinct modes of gene regulation and widespread enhancer reprogramming by the oncogenic fusion protein EWS-FLI1. Cell Rep.

[64] Cheung KL, Kim C, Zhou MM (2021). The Functions of BET Proteins in Gene Transcription of Biology and Diseases. Front Mol Biosci.

[65] Li M, Huang H, Li L, He C, Zhu L, Guo H (2021). Core transcription regulatory circuitry orchestrates corneal epithelial homeostasis. Nat Commun.

[66] Qian FC, Li XC, Guo JC, Zhao JM, Li YY, Tang ZD (2019). SEanalysis: a web tool for super-enhancer associated regulatory analysis. Nucleic Acids Res.

[67] Gluck C, Glathar A, Tsompana M, Nowak N, Garrett-Sinha LA, Buck MJ (2019). Molecular dissection of the oncogenic role of ETS1 in the mesenchymal subtypes of head and neck squamous cell carcinoma. PLoS Genet.

[68] Lee KW, Yeo SY, Gong JR, Koo OJ, Sohn I, Lee WY (2022). PRRX1 is a master transcription factor of stromal fibroblasts for myofibroblastic lineage progression. Nat Commun.

[69] Zhang T, Song X, Zhang Z, Mao Q, Xia W, Xu L (2020). Aberrant super-enhancer landscape reveals core transcriptional regulatory circuitry in lung adenocarcinoma. Oncogenesis.

[70] Durbin AD, Zimmerman MW, Dharia NV, Abraham BJ, Iniguez AB, Weichert-Leahey N (2018). Selective gene dependencies in MYCN-amplified neuroblastoma include the core transcriptional regulatory circuitry. Nat Genet.

[71] Ran L, Chen Y, Sher J, Wong EWP, Murphy D, Zhang JQ (2018). FOXF1 Defines the Core-Regulatory Circuitry in Gastrointestinal Stromal Tumor. Cancer Discov.

[72] Lin C, Yuan H, Wang W, Zhu Z, Lu Y, Wang J (2020). Importance of PNO1 for growth and survival of urinary bladder carcinoma: Role in core-regulatory circuitry. J Cell Mol Med.

[73] Chen Y, Xu L, Lin RY, Müschen M, Koeffler HP (2020). Core transcriptional regulatory circuitries in cancer. Oncogene.

[74] Jiang Y, Jiang Y, Li C, Zhang Y, Dakle P, Kaur H (2020). TP63, SOX2, and KLF5 Establish a Core Regulatory Circuitry That Controls Epigenetic and Transcription Patterns in Esophageal Squamous Cell Carcinoma Cell Lines. Gastroenterology.

[75] Lu B, Zou C, Yang M, He Y, He J, Zhang C (2021). Pharmacological Inhibition of Core Regulatory Circuitry Liquid-liquid Phase Separation Suppresses Metastasis and Chemoresistance in Osteosarcoma. Adv Sci (Weinh).

[76] Chen L, Huang M, Plummer J, Pan J, Jiang Y, Yang Q (2020). Master transcription factors form interconnected circuitry and orchestrate transcriptional networks in oesophageal adenocarcinoma. Gut.

[77] Baumgart SJ, Nevedomskaya E, Haendler B. Dysregulated Transcriptional Control in Prostate Cancer. Int J Mol Sci. 2019;20(12):2883. 10.3390/ijms20122883 PMC6627928 31200487

[78] Donati B, Lorenzini E, Ciarrocchi A (2018). BRD4 and Cancer: going beyond transcriptional regulation. Mol Cancer.

[79] Parua PK, Kalan S, Benjamin B, Sansó M, Fisher RP (2020). Distinct Cdk9-phosphatase switches act at the beginning and end of elongation by RNA polymerase II. Nat Commun.

[80] Lambert S, Jolma A, Campitelli L, Das P, Yin Y, Albu M (2018). The Human Transcription Factors. Cell.

[81] Wang C, Zhang L, Ke L, Ding W, Jiang S, Li D (2020). Primary effusion lymphoma enhancer connectome links super-enhancers to dependency factors. Nat Commun.

[82] Ceribelli M, Hou ZE, Kelly PN, Huang DW, Wright G, Ganapathi K (2016). A Druggable TCF4- and BRD4-Dependent Transcriptional Network Sustains Malignancy in Blastic Plasmacytoid Dendritic Cell Neoplasm. Cancer Cell.

[83] Nguyen DT, Yang W, Renganathan A, Weimholt C, Angappulige DH, Nguyen T (2022). Acetylated HOXB13 Regulated Super Enhancer Genes Define Therapeutic Vulnerabilities of Castration-Resistant Prostate Cancer. Clin Cancer Res.

[84] Peng JY, Cai DK, Zeng RL, Zhang CY, Li GC, Chen SF (2022). Upregulation of Superenhancer-Driven LncRNA FASRL by USF1 Promotes De Novo Fatty Acid Biosynthesis to Exacerbate Hepatocellular Carcinoma. Adv Sci (Weinh).

[85] Jiang Y, Jiang YY, Xie JJ, Mayakonda A, Hazawa M, Chen L (2018). Co-activation of super-enhancer-driven CCAT1 by TP63 and SOX2 promotes squamous cancer progression. Nat Commun.

[86] Han J, Meng J, Chen S, Wang X, Yin S, Zhang Q (2019). YY1 Complex Promotes Quaking Expression via Super-Enhancer Binding during EMT of Hepatocellular Carcinoma. Can Res.

[87] Marques JG, Gryder BE, Pavlovic B, Chung Y, Ngo QA, Frommelt F, et al. NuRD subunit CHD4 regulates super-enhancer accessibility in rhabdomyosarcoma and represents a general tumor dependency. Elife. 2020;9:e54993. 10.7554/eLife.54993 PMC7438112 32744500

[88] Lo-Coco F, Avvisati G, Vignetti M, Thiede C, Orlando S, Iacobelli S (2013). Retinoic acid and arsenic trioxide for acute promyelocytic leukemia. N Engl J Med.

[89] Chen R, Zhang M, Zhou Y, Guo W, Yi M, Zhang Z (2020). The application of histone deacetylases inhibitors in glioblastoma. J Exp Clin Cancer Res.

[90] Wang Z, Zang C, Rosenfeld J, Schones D, Barski A, Cuddapah S (2008). Combinatorial patterns of histone acetylations and methylations in the human genome. Nat Genet.

[91] Kumaraswamy A, Welker Leng KR, Westbrook TC, Yates JA, Zhao SG, Evans CP (2021). Recent Advances in Epigenetic Biomarkers and Epigenetic Targeting in Prostate Cancer. Eur Urol.

[92] Jia Y, Zhou J, Tan T, Chung T, Chen Y, Chooi J (2022). HJURPSuper Enhancer-Mediated Upregulation of Promotes Growth and Survival of t(4;14)-Positive Multiple Myeloma. Can Res.

[93] Darvishi E, Slemmons K, Wan Z, Mitra S, Hou X, Hugues Parmentier J (2020). Molecular mechanisms of Guadecitabine induced FGFR4 down regulation in alveolar rhabdomyosarcomas. Neoplasia (New York, NY).

[94] Andricovich J, Perkail S, Kai Y, Casasanta N, Peng W, Tzatsos A (2018). Loss of KDM6A Activates Super-Enhancers to Induce Gender-Specific Squamous-like Pancreatic Cancer and Confers Sensitivity to BET Inhibitors. Cancer Cell.

[95] Dai W, Wu J, Peng X, Hou W, Huang H, Cheng Q (2022). CDK12 orchestrates super-enhancer-associated CCDC137 transcription to direct hepatic metastasis in colorectal cancer. Clin Transl Med.

[96] Chen Q, Yang B, Liu X, Zhang XD, Zhang L, Liu T (2022). Histone acetyltransferases CBP/p300 in tumorigenesis and CBP/p300 inhibitors as promising novel anticancer agents. Theranostics.

[97] Zhang T, Xia W, Song X, Mao Q, Huang X, Chen B (2022). Super-enhancer hijacking LINC01977 promotes malignancy of early-stage lung adenocarcinoma addicted to the canonical TGF-β/SMAD3 pathway. J Hematol Oncol.

[98] Groselj B, Sharma N, Hamdy F, Kerr M, Kiltie A (2013). Histone deacetylase inhibitors as radiosensitisers: effects on DNA damage signalling and repair. Br J Cancer.

[99] Brilli LL, Swanhart LM, de Caestecker MP, Hukriede NA (2013). HDAC inhibitors in kidney development and disease. Pediatr Nephrol.

[100] Natsume A, Hirano M, Ranjit M, Aoki K, Wakabayashi T (2019). Aberrant Transcriptional Regulation of Super-enhancers by RET Finger Protein-histone Deacetylase 1 Complex in Glioblastoma: Chemoresistance to Temozolomide. Neurol Med Chir (Tokyo).

[101] Gryder BE, Wu L, Woldemichael GM, Pomella S, Quinn TR, Park PMC (2019). Chemical genomics reveals histone deacetylases are required for core regulatory transcription. Nat Commun.

[102] Caslini C, Hong S, Ban YJ, Chen XS, Ince TA (2019). HDAC7 regulates histone 3 lysine 27 acetylation and transcriptional activity at super-enhancer-associated genes in breast cancer stem cells. Oncogene.

[103] Pinz S, Unser S, Rascle A (2016). Signal transducer and activator of transcription STAT5 is recruited to c-Myc super-enhancer. BMC Mol Biol.

[104] Fontanals-Cirera B, Hasson D, Vardabasso C, Di Micco R, Agrawal P, Chowdhury A (2017). Harnessing BET Inhibitor Sensitivity Reveals AMIGO2 as a Melanoma Survival Gene. Mol Cell.

[105] Murakami S, Li R, Nagari A, Chae M, Camacho CV, Kraus WL (2019). Distinct Roles for BET Family Members in Estrogen Receptor α Enhancer Function and Gene Regulation in Breast Cancer Cells. Mol Cancer Res.

[106] Wang X, Kutschat AP, Yamada M, Prokakis E, Böttcher P, Tanaka K (2021). Bromodomain protein BRDT directs ΔNp63 function and super-enhancer activity in a subset of esophageal squamous cell carcinomas. Cell Death Differ.

[107] Liu J, Duan Z, Guo W, Zeng L, Wu Y, Chen Y (2018). Targeting the BRD4/FOXO3a/CDK6 axis sensitizes AKT inhibition in luminal breast cancer. Nat Commun.

[108] Devaiah B, Gegonne A, Singer D (2016). Bromodomain 4: a cellular Swiss army knife. J Leukoc Biol.

[109] Dong J, Li J, Li Y, Ma Z, Yu Y, Wang CY (2021). Transcriptional super-enhancers control cancer stemness and metastasis genes in squamous cell carcinoma. Nat Commun.

[110] Liu B, Liu X, Han L, Chen X, Wu X, Wu J, et al. BRD4-directed super-enhancer organization of transcription repression programs links to chemotherapeutic efficacy in breast cancer. Proc Natl Acad Sci U S A. 2022;119(6):e2109133119. 10.1073/pnas.2109133119 PMC8832982 35105803

[111] Kim EJ, Liu P, Zhang S, Donahue K, Wang Y, Schehr JL (2021). BAF155 methylation drives metastasis by hijacking super-enhancers and subverting anti-tumor immunity. Nucleic Acids Res.

[112] Zhang S, O'Regan R, Xu W (2020). The emerging role of mediator complex subunit 12 in tumorigenesis and response to chemotherapeutics. Cancer.

[113] Chen W, Roeder R (2011). Mediator-dependent nuclear receptor function. Semin Cell Dev Biol.

[114] Lovén J, Hoke H, Lin C, Lau A, Orlando D, Vakoc C (2013). Selective inhibition of tumor oncogenes by disruption of super-enhancers. Cell.

[115] Zhou B, Wang L, Zhang S, Bennett BD, He F, Zhang Y (2016). INO80 governs superenhancer-mediated oncogenic transcription and tumor growth in melanoma. Genes Dev.

[116] Bian E, Chen X, Cheng L, Cheng M, Chen Z, Yue X (2021). Super-enhancer-associated TMEM44-AS1 aggravated glioma progression by forming a positive feedback loop with Myc. J Exp Clin Cancer Res.

[117] Russo JW, Nouri M, Balk SP (2019). Androgen Receptor Interaction with Mediator Complex Is Enhanced in Castration-Resistant Prostate Cancer by CDK7 Phosphorylation of MED1. Cancer Discov.

[118] Baumgart SJ, Nevedomskaya E, Lesche R, Newman R, Mumberg D, Haendler B (2020). Darolutamide antagonizes androgen signaling by blocking enhancer and super-enhancer activation. Mol Oncol.

[119] Kuuluvainen E, Domènech-Moreno E, Niemelä E, Mäkelä T. Depletion of Mediator Kinase Module Subunits Represses Superenhancer-Associated Genes in Colon Cancer Cells. Mol Cell Biol. 2018;38(11):e00573–17. 10.1128/MCB.00573-17 PMC5954191 29507187

[120] Tsang FH, Law CT, Tang TC, Cheng CL, Chin DW, Tam WV (2019). Aberrant Super-Enhancer Landscape in Human Hepatocellular Carcinoma. Hepatology.

[121] Sena LA, Kumar R, Sanin DE, Thompson EA, Rosen DM, Dalrymple SL, et al. Androgen receptor activity in prostate cancer dictates efficacy of bipolar androgen therapy through MYC. J Clin Invest. 2022;132(23):e162396. 10.1172/JCI162396 PMC9711876 36194476

[122] Malumbres M (2014). Cyclin-dependent kinases. Genome Biol.

[123] Malumbres M, Barbacid M (2009). Cell cycle, CDKs and cancer: a changing paradigm. Nat Rev Cancer.

[124] Jonkers I, Lis J (2015). Getting up to speed with transcription elongation by RNA polymerase II. Nat Rev Mol Cell Biol.

[125] Kwiatkowski N, Zhang T, Rahl PB, Abraham BJ, Reddy J, Ficarro SB (2014). Targeting transcription regulation in cancer with a covalent CDK7 inhibitor. Nature.

[126] Yuan J, Jiang YY, Mayakonda A, Huang M, Ding LW, Lin H (2017). Super-Enhancers Promote Transcriptional Dysregulation in Nasopharyngeal Carcinoma. Cancer Res.

[127] Lin L, Huang M, Shi X, Mayakonda A, Hu K, Jiang Y (2019). Super-enhancer-associated MEIS1 promotes transcriptional dysregulation in Ewing sarcoma in co-operation with EWS-FLI1. Nucleic Acids Res.

[128] Egloff S (2021). CDK9 keeps RNA polymerase II on track. Cell Mol Life Sci.

[129] Sharifnia T, Wawer MJ, Chen T, Huang QY, Weir BA, Sizemore A (2019). Small-molecule targeting of brachyury transcription factor addiction in chordoma. Nat Med.

[130] Hanahan D (2022). Hallmarks of Cancer: New Dimensions. Cancer Discov.

[131] Hosoi H, Niibori-Nambu A, Nah G, Bahirvani A, Mok M, Sanda T (2021). Super-enhancers for RUNX3 are required for cell proliferation in EBV-infected B cell lines. Gene.

[132] Jiang Y, Lin D, Mayakonda A, Hazawa M, Ding L, Chien W (2017). Targeting super-enhancer-associated oncogenes in oesophageal squamous cell carcinoma. Gut.

[133] Karpel-Massler G, Ishida C, Zhang Y, Halatsch M, Westhoff M, Siegelin M (2017). Targeting intrinsic apoptosis and other forms of cell death by BH3-mimetics in glioblastoma. Expert Opin Drug Discov.

[134] Ning H, Huang S, Lei Y, Zhi R, Yan H, Jin J (2022). Enhancer decommissioning by MLL4 ablation elicits dsRNA-interferon signaling and GSDMD-mediated pyroptosis to potentiate anti-tumor immunity. Nat Commun.

[135] Lugano R, Ramachandran M, Dimberg A (2020). Tumor angiogenesis: causes, consequences, challenges and opportunities. Cell Mol Life Sci.

[136] Katanasaka Y, Kodera Y, Kitamura Y, Morimoto T, Tamura T, Koizumi F (2013). Epidermal growth factor receptor variant type III markedly accelerates angiogenesis and tumor growth via inducing c-myc mediated angiopoietin-like 4 expression in malignant glioma. Mol Cancer.

[137] Moran J, Kim H, Li Z, Moreno C (2019). SOX4 regulates invasion of bladder cancer cells via repression of WNT5a. Int J Oncol.

[138] Zhou J, Wang D, Tang D, Huang W (2020). Abnormal Activations of Super-Enhancers Enhance the Carcinogenicity in Lung Adenocarcinoma. Cancer Manag Res.

[139] Seidl C (2020). Targets for Therapy of Bladder Cancer. Semin Nucl Med.

[140] Zhang J, Liu W, Zou C, Zhao Z, Lai Y, Shi Z (2020). Targeting Super-Enhancer-Associated Oncogenes in Osteosarcoma with THZ2, a Covalent CDK7 Inhibitor. Clin Cancer Res.

[141] Liao Y, Feng Y, Shen J, Gao Y, Cote G, Choy E (2016). Clinical and biological significance of PIM1 kinase in osteosarcoma. J Orthop Res.

[142] Zhang J, Zhou Y, Yue W, Zhu Z, Wu X, Yu S (2022). Super-enhancers conserved within placental mammals maintain stem cell pluripotency. Proc Natl Acad Sci USA.

[143] Cui S, Wu Q, Liu M, Su M, Liu S, Shao L (2021). EphA2 super-enhancer promotes tumor progression by recruiting FOSL2 and TCF7L2 to activate the target gene EphA2. Cell Death Dis.

[144] Jin H, Wang C, Jin G, Ruan H, Gu D, Wei L (2017). Regulator of Calcineurin 1 Gene Isoform 4, Down-regulated in Hepatocellular Carcinoma, Prevents Proliferation, Migration, and Invasive Activity of Cancer Cells and Metastasis of Orthotopic Tumors by Inhibiting Nuclear Translocation of NFAT1. Gastroenterology.

[145] Williams E, Gao D, Redfern A, Thompson E (2019). Controversies around epithelial-mesenchymal plasticity in cancer metastasis. Nat Rev Cancer.

[146] Dongre A, Weinberg R (2019). New insights into the mechanisms of epithelial-mesenchymal transition and implications for cancer. Nat Rev Mol Cell Biol.

[147] Lamouille S, Xu J, Derynck R (2014). Molecular mechanisms of epithelial-mesenchymal transition. Nat Rev Mol Cell Biol.

[148] Yamamoto-Fukuda T, Akiyama N, Kojima H (2021). Super-enhancer Acquisition Drives FOXC2 Expression in Middle Ear Cholesteatoma. J Assoc Res Otolaryngol.

[149] Zhang C, Wei S, Sun W, Teng K, Dai M, Wang F (2020). Super-enhancer-driven AJUBA is activated by TCF4 and involved in epithelial-mesenchymal transition in the progression of Hepatocellular Carcinoma. Theranostics.

[150] Zhu X, Zhang T, Zhang Y, Chen H, Shen J, Jin X (2020). A super-enhancer controls TGF- β signaling in pancreatic cancer through downregulation of TGFBR2. Cell Signal.

[151] Mooso B, Vinall R, Mudryj M, Yap S, deVere WR, Ghosh P (2015). The role of EGFR family inhibitors in muscle invasive bladder cancer: a review of clinical data and molecular evidence. J Urol.

[152] Clara JA, Monge C, Yang Y, Takebe N (2020). Targeting signalling pathways and the immune microenvironment of cancer stem cells - a clinical update. Nat Rev Clin Oncol.

[153] Clarke M (2019). Clinical and Therapeutic Implications of Cancer Stem Cells. N Engl J Med.

[154] Gimple R, Kidwell R, Kim L, Sun T, Gromovsky A, Wu Q (2019). Glioma Stem Cell-Specific Superenhancer Promotes Polyunsaturated Fatty-Acid Synthesis to Support EGFR Signaling. Cancer Discov.

[155] Zhang M, Hoyle R, Ma Z, Sun B, Cai W, Cai H (2021). FOSL1 promotes metastasis of head and neck squamous cell carcinoma through super-enhancer-driven transcription program. Mol Ther.

[156] Ousman SS, Kubes P (2012). Immune surveillance in the central nervous system. Nat Neurosci.

[157] Teppo S, Laukkanen S, Liuksiala T, Nordlund J, Oittinen M, Teittinen K (2016). Genome-wide repression of eRNA and target gene loci by the ETV6-RUNX1 fusion in acute leukemia. Genome Res.

[158] Casey S, Tong L, Li Y, Do R, Walz S, Fitzgerald K (2016). MYC regulates the antitumor immune response through CD47 and PD-L1. Science (New York, NY).

[159] Xu Y, Wu Y, Zhang S, Ma P, Jin X, Wang Z (2019). A Tumor-Specific Super-Enhancer Drives Immune Evasion by Guiding Synchronous Expression of PD-L1 and PD-L2. Cell Rep.

[160] Zhang C, Chen L, Liu Y, Huang J, Liu A, Xu Y (2021). Downregulated METTL14 accumulates BPTF that reinforces super-enhancers and distal lung metastasis via glycolytic reprogramming in renal cell carcinoma. Theranostics.

[161] Tateishi K, Iafrate A, Ho Q, Curry W, Batchelor T, Flaherty K (2016). Myc-Driven Glycolysis Is a Therapeutic Target in Glioblastoma. Clin Cancer Res.

[162] Wang J, Wang H, Li Z, Wu Q, Lathia J, McLendon R (2008). c-Myc is required for maintenance of glioma cancer stem cells. PLoS One.

[163] Alam H, Tang M, Maitituoheti M, Dhar SS, Kumar M, Han CY (2020). KMT2D Deficiency Impairs Super-Enhancers to Confer a Glycolytic Vulnerability in Lung Cancer. Cancer Cell.

[164] Wu F, Xu L, Tu Y, Cheung O, Szeto L, Mok M (2022). Sirtuin 7 super-enhancer drives epigenomic reprogramming in hepatocarcinogenesis. Cancer Lett.

[165] Djebali S, Davis C, Merkel A, Dobin A, Lassmann T, Mortazavi A (2012). Landscape of transcription in human cells. Nature.

[166] Pan J, Hu S, Ren X, Hu H, Deng X, Yu B (2022). Whole-Transcriptome Profiling and circRNA-miRNA-mRNA Regulatory Networks in B-Cell Development. Front Immunol.

[167] Wang Y, Nie H, He X, Liao Z, Zhou Y, Zhou J (2020). The emerging role of super enhancer-derived noncoding RNAs in human cancer. Theranostics.

[168] Kopp F, Mendell JT (2018). Functional Classification and Experimental Dissection of Long Noncoding RNAs. Cell.

[169] Li C, Yang J, Liu C, Wang X, Zhang L (2020). Long non-coding RNAs in hepatocellular carcinoma: Ordering of the complicated lncRNA regulatory network and novel strategies for HCC clinical diagnosis and treatment. Pharmacol Res.

[170] Tan Y, Li Y, Tang F (2020). Oncogenic seRNA functional activation: a novel mechanism of tumorigenesis. Mol Cancer.

[171] Melo C, Drost J, Wijchers P, van de Werken H, de Wit E, Oude Vrielink J (2013). eRNAs are required for p53-dependent enhancer activity and gene transcription. Mol Cell.

[172] Wang X, Zhang R, Wu S, Shen L, Ke M, Ouyang Y (2020). Super-Enhancer LncRNA LINC00162 Promotes Progression of Bladder Cancer. iScience.

[173] Xiang JF, Yin QF, Chen T, Zhang Y, Zhang XO, Wu Z (2014). Human colorectal cancer-specific CCAT1-L lncRNA regulates long-range chromatin interactions at the MYC locus. Cell Res.

[174] Das S, Senapati P, Chen Z, Reddy M, Ganguly R, Lanting L (2017). Regulation of angiotensin II actions by enhancers and super-enhancers in vascular smooth muscle cells. Nat Commun.

[175] Peng L, Jiang B, Yuan X, Qiu Y, Peng J, Huang Y (2019). Super-Enhancer-Associated Long Noncoding RNA HCCL5 Is Activated by ZEB1 and Promotes the Malignancy of Hepatocellular Carcinoma. Cancer Res.

[176] Zhang Y, Huang YX, Wang DL, Yang B, Yan HY, Lin LH (2020). LncRNA DSCAM-AS1 interacts with YBX1 to promote cancer progression by forming a positive feedback loop that activates FOXA1 transcription network. Theranostics.

[177] Wang QY, Peng L, Chen Y, Liao LD, Chen JX, Li M (2020). Characterization of super-enhancer-associated functional lncRNAs acting as ceRNAs in ESCC. Mol Oncol.

[178] Xie JJ, Jiang YY, Jiang Y, Li CQ, Lim MC, An O (2018). Super-Enhancer-Driven Long Non-Coding RNA LINC01503, Regulated by TP63, Is Over-Expressed and Oncogenic in Squamous Cell Carcinoma. Gastroenterology.

[179] Di Agostino S, Valenti F, Sacconi A, Fontemaggi G, Pallocca M, Pulito C (2018). Long Non-coding MIR205HG Depletes Hsa-miR-590-3p Leading to Unrestrained Proliferation in Head and Neck Squamous Cell Carcinoma. Theranostics.

[180] Liang W, Shi C, Hong W, Li P, Zhou X, Fu W (2021). Super-enhancer-driven lncRNA-DAW promotes liver cancer cell proliferation through activation of Wnt/β-catenin pathway. Mol Ther Nucleic Acids.

[181] Xu S, Wan L, Yin H, Xu H, Zheng W, Shen M (2017). Long Noncoding RNA Linc00152 Functions as a Tumor Propellant in Pan-Cancer. Cell Physiol Biochem.

[182] Lin X, Spindler TJ, de Souza Fonseca MA, Corona RI, Seo JH, Dezem FS (2019). Super-Enhancer-Associated LncRNA UCA1 Interacts Directly with AMOT to Activate YAP Target Genes in Epithelial Ovarian Cancer. iScience.

[183] Bartel D (2004). MicroRNAs: genomics, biogenesis, mechanism, and function. Cell.

[184] Balzeau J, Menezes M, Cao S, Hagan J (2017). The LIN28/let-7 Pathway in Cancer. Front Genet.

[185] Xiao M, Li J, Li W, Wang Y, Wu F, Xi Y (2017). MicroRNAs activate gene transcription epigenetically as an enhancer trigger. RNA Biol.

[186] Li W, Yang S, Xu P, Zhang D, Tong Y, Chen L (2022). SARS-CoV-2 RNA elements share human sequence identity and upregulate hyaluronan via NamiRNA-enhancer network. EBioMedicine.

[187] Miyakawa K, Miyashita N, Horie M, Terasaki Y, Tanaka H, Urushiyama H (2022). ASCL1 regulates super-enhancer-associated miRNAs to define molecular subtypes of small cell lung cancer. Cancer Sci.

[188] Anandagoda N, Willis JC, Hertweck A, Roberts LB, Jackson I, Gökmen MR (2019). microRNA-142-mediated repression of phosphodiesterase 3B critically regulates peripheral immune tolerance. J Clin Invest.

[189] Hnisz D, Abraham BJ, Lee TI, Lau A, Saint-André V, Sigova AA (2013). Super-enhancers in the control of cell identity and disease. Cell.

[190] Suzuki HI, Young RA, Sharp PA (2017). Super-Enhancer-Mediated RNA Processing Revealed by Integrative MicroRNA Network Analysis. Cell.

[191] An J, Xu J, Li J, Jia S, Li X, Lu Y (2017). HistoneH3 demethylase JMJD2A promotes growth of liver cancer cells through up-regulating miR372. Oncotarget.

[192] Nasser MW, Datta J, Nuovo G, Kutay H, Motiwala T, Majumder S (2018). Down-regulation of micro-RNA-1 (miR-1) in lung cancer. Suppression of tumorigenic property of lung cancer cells and their sensitization to doxorubicin-induced apoptosis by miR-1. J Biol Chem.

[193] Duan Q, Mao X, Xiao Y, Liu Z, Wang Y, Zhou H (2016). Super enhancers at the miR-146a and miR-155 genes contribute to self-regulation of inflammation. Biochem Biophys Acta.

[194] Al-Haidari A, Algaber A, Madhi R, Syk I, Thorlacius H (2018). MiR-155-5p controls colon cancer cell migration via post-transcriptional regulation of Human Antigen R (HuR). Cancer Lett.

[195] Garo L, Ajay A, Fujiwara M, Gabriely G, Raheja R, Kuhn C (2021). MicroRNA-146a limits tumorigenic inflammation in colorectal cancer. Nat Commun.

[196] Li X, Yang L, Chen L (2018). The Biogenesis, Functions, and Challenges of Circular RNAs. Mol Cell.

[197] Zhang Z, Yang T, Xiao J (2018). Circular RNAs: Promising Biomarkers for Human Diseases. EBioMedicine.

[198] Lei M, Zheng G, Ning Q, Zheng J, Dong D (2020). Translation and functional roles of circular RNAs in human cancer. Mol Cancer.

[199] Huang S, Li X, Zheng H, Si X, Li B, Wei G (2019). Loss of Super-Enhancer-Regulated circRNA Nfix Induces Cardiac Regeneration After Myocardial Infarction in Adult Mice. Circulation.

[200] Ding C, Wu Z, You H, Ge H, Zheng S, Lin Y (2019). CircNFIX promotes progression of glioma through regulating miR-378e/RPN2 axis. J Exp Clin Cancer Res.

[201] Li X, Tang Z, Peng L, Li Y, Qian F, Zhao J (2020). Integrative Epigenomic Analysis of Transcriptional Regulation of Human CircRNAs. Front Genet.

[202] Khan A, Zhang X (2016). dbSUPER: a database of super-enhancers in mouse and human genome. Nucleic Acids Res.

[203] Wei Y, Zhang S, Shang S, Zhang B, Li S, Wang X (2016). SEA: a super-enhancer archive. Nucleic Acids Res.

[204] Wang T, Zhang H, Zhou Y, Shi J (2021). Extrachromosomal circular DNA: a new potential role in cancer progression. J Transl Med.

[205] Luo L, Gribskov M, Wang S. Bibliometric review of ATAC-Seq and its application in gene expression. Brief Bioinformatics. 2022;23(3):bbac061. 10.1093/bib/bbac061 PMC9116206 35255493

[206] Li Q, Lin X, Yu Y, Chen L, Hu Q, Chen M (2021). Genome-wide profiling in colorectal cancer identifies PHF19 and TBC1D16 as oncogenic super enhancers. Nat Commun.

[207] Jia R, Chai P, Zhang H, Fan X (2017). Novel insights into chromosomal conformations in cancer. Mol Cancer.

[208] Mei S, Meyer CA, Zheng R, Qin Q, Wu Q, Jiang P (2017). Cistrome Cancer: A Web Resource for Integrative Gene Regulation Modeling in Cancer. Cancer Res.

[209] Chen C, Zhou D, Gu Y, Wang C, Zhang M, Lin X (2020). SEA version 3.0: a comprehensive extension and update of the Super-Enhancer archive. Nucleic Acids Res.

[210] Jiang Y, Qian F, Bai X, Liu Y, Wang Q, Ai B (2019). SEdb: a comprehensive human super-enhancer database. Nucleic Acids Res.

[211] Guo Z, Xie C, Li K, Zhai X, Cai G, Yang X, et al. SELER: a database of super-enhancer-associated lncRNA- directed transcriptional regulation in human cancers. Database. 2019;2019:baz027. 10.1093/database/baz027 PMC6390648 30806704

[212] Tang Z, Li X, Zhao J, Qian F, Feng C, Li Y (2019). TRCirc: a resource for transcriptional regulation information of circRNAs. Brief Bioinform.

[213] Lanza D, Gaspero A, Lorenzo I, Liao L, Zheng P, Wang Y (2018). Comparative analysis of single-stranded DNA donors to generate conditional null mouse alleles. BMC Biol.

[214] Huang H, Hu J, Maryam A, Huang Q, Zhang Y, Ramakrishnan S (2021). Defining super-enhancer landscape in triple-negative breast cancer by multiomic profiling. Nat Commun.

[215] Jia Y, Chng WJ, Zhou J (2019). Super-enhancers: critical roles and therapeutic targets in hematologic malignancies. J Hematol Oncol.

[216] Tseng H, Hwang Y, Yeh K, Chang J, Chen Y, Yu H (2009). Reduced expression of C/EBP alpha protein in hepatocellular carcinoma is associated with advanced tumor stage and shortened patient survival. J Cancer Res Clin Oncol.

[217] Baldauf M, Orth M, Dallmayer M, Marchetto A, Gerke J, Rubio R (2018). Robust diagnosis of Ewing sarcoma by immunohistochemical detection of super-enhancer-driven EWSR1-ETS targets. Oncotarget.

[218] Xing R, Zhou Y, Yu J, Yu Y, Nie Y, Luo W (2019). Whole-genome sequencing reveals novel tandem-duplication hotspots and a prognostic mutational signature in gastric cancer. Nat Commun.

[219] Saito T, Asai S, Tanaka N, Nohata N, Minemura C, Koma A, et al. Genome-Wide Super-Enhancer-Based Analysis: Identification of Prognostic Genes in Oral Squamous Cell Carcinoma. Int J Mol Sci. 2022;23(16):9154. 10.3390/ijms23169154 PMC9409227 36012427

[220] Li G, Qu Q, Qi T, Teng X, Zhu H, Wang J (2021). Super-enhancers: a new frontier for epigenetic modifiers in cancer chemoresistance. J Exp Clin Cancer Res.

[221] Kim J, Wang C, de Sabando AR, Cole HL, Huang TJ, Yang J (2019). The Novel Small-Molecule SR18662 Efficiently Inhibits the Growth of Colorectal Cancer In Vitro and In Vivo. Mol Cancer Ther.

[222] Chen Z, Wu Q, Ding Y, Zhou W, Liu R, Chen H (2017). YD277 Suppresses Triple-Negative Breast Cancer Partially Through Activating the Endoplasmic Reticulum Stress Pathway. Theranostics.

[223] Fang L, Zhu Q, Neuenschwander M, Specker E, Wulf-Goldenberg A, Weis W (2016). A Small-Molecule Antagonist of the β-Catenin/TCF4 Interaction Blocks the Self-Renewal of Cancer Stem Cells and Suppresses Tumorigenesis. Can Res.

[224] Ding T, Zhu Y, Jin H, Zhang P, Guo J, Zheng J (2021). Circular RNA circ_0057558 Controls Prostate Cancer Cell Proliferation Through Regulating miR-206/USP33/c-Myc Axis. Front Cell Dev Biol.

[225] Boike L, Cioffi A, Majewski F, Co J, Henning N, Jones M (2021). Discovery of a Functional Covalent Ligand Targeting an Intrinsically Disordered Cysteine within MYC. Cell Chem Biol.

[226] Yu X, Yang F, Jiang H, Fan L (2020). RGFP966 Suppresses Tumor Growth and Migration Through Inhibition of EGFR Expression in Hepatocellular Carcinoma Cells in vitro. Drug Des Dev Ther.

[227] Song L, Bretz A, Gravemeyer J, Spassova I, Muminova S, Gambichler T (2021). The HDAC Inhibitor Domatinostat Promotes Cell-Cycle Arrest, Induces Apoptosis, and Increases Immunogenicity of Merkel Cell Carcinoma Cells. J Invest Dermatol.

[228] Sanchez G, Richmond P, Bunker E, Karman S, Azofeifa J, Garnett A (2018). Genome-wide dose-dependent inhibition of histone deacetylases studies reveal their roles in enhancer remodeling and suppression of oncogenic super-enhancers. Nucleic Acids Res.

[229] Wang L, Chang J, Varghese D, Dellinger M, Kumar S, Best A (2013). A small molecule modulates Jumonji histone demethylase activity and selectively inhibits cancer growth. Nat Commun.

[230] Mai J, Gu J, Liu Y, Liu X, Sai K, Chen Z (2019). Negative regulation of miR-1275 by H3K27me3 is critical for glial induction of glioblastoma cells. Mol Oncol.

[231] Zhang W, Cheng J, Diao P, Wang D, Zhang W, Jiang H (2020). Therapeutically targeting head and neck squamous cell carcinoma through synergistic inhibition of LSD1 and JMJD3 by TCP and GSK-J1. Br J Cancer.

[232] Akinsiku O, Soremekun O, Olotu F, Soliman M (2020). Exploring the Role of Asp1116 in Selective Drug Targeting of CREBcAMP- Responsive Element-binding Protein Implicated in Prostate Cancer. Comb Chem High Throughput Screen.

[233] Kang S, Bae H, Kwon W, Kim T, Kim K, Park S (2021). Inhibition of the bromodomain and extra-terminal family of epigenetic regulators as a promising therapeutic approach for gastric cancer. Cell Oncol (Dordr).

[234] Wang L, Xu M, Kao C, Tsai S, Tsai M (2020). Small molecule JQ1 promotes prostate cancer invasion via BET-independent inactivation of FOXA1. J Clin Investig.

[235] Yokoyama Y, Zhu H, Lee J, Kossenkov A, Wu S, Wickramasinghe J (2016). BET Inhibitors Suppress ALDH Activity by Targeting ALDH1A1 Super-Enhancer in Ovarian Cancer. Can Res.

[236] Shin HY (2018). Targeting Super-Enhancers for Disease Treatment and Diagnosis. Mol Cells.

[237] Meng W, Wang J, Wang B, Liu F, Li M, Zhao Y (2018). CDK7 inhibition is a novel therapeutic strategy against GBM both in vitro and in vivo. Cancer Manag Res.

[238] Shao Y, Li Y, Hsu H, Lin H, Wang H, Wo R, et al. Potent Activity of Composite Cyclin Dependent Kinase Inhibition against Hepatocellular Carcinoma. Cancers. 2019;11(10):1433. 10.3390/cancers11101433 PMC6827105 31561409

[239] Chen L, Hung L, Tsai K, Pan Y, Tsai Y, Li Y (2008). Wogonin, a bioactive flavonoid in herbal tea, inhibits inflammatory cyclooxygenase-2 gene expression in human lung epithelial cancer cells. Mol Nutr Food Res.

[240] Chen Y, Shen S, Lee W, Lin H, Ko C, Shih C (2002). Wogonin and fisetin induction of apoptosis through activation of caspase 3 cascade and alternative expression of p21 protein in hepatocellular carcinoma cells SK-HEP-1. Arch Toxicol.

[241] Liao X, Hong Y, Mao Y, Chen N, Wang Q, Wang Z (2020). SPH3643: A novel cyclin-dependent kinase 4/6 inhibitor with good anticancer efficacy and strong blood-brain barrier permeability. Cancer Sci.

[242] Lei H, Wang Z, Jiang D, Liu F, Liu M, Lei X (2021). CRISPR screening identifies CDK12 as a conservative vulnerability of prostate cancer. Cell Death Dis.

[243] Witt O, Deubzer H, Milde T, Oehme I (2009). HDAC family: What are the cancer relevant targets?. Cancer Lett.

[244] Nguyen T, Zhang Y, Shang E, Shu C, Torrini C, Zhao J (2020). HDAC inhibitors elicit metabolic reprogramming by targeting super-enhancers in glioblastoma models. J Clin Investig.

[245] Crisanti M, Wallace A, Kapoor V, Vandermeers F, Dowling M, Pereira L (2009). The HDAC inhibitor panobinostat (LBH589) inhibits mesothelioma and lung cancer cells in vitro and in vivo with particular efficacy for small cell lung cancer. Mol Cancer Ther.

[246] Cochran A, Conery A, Sims R (2019). Bromodomains: a new target class for drug development. Nat Rev Drug Discov.

[247] Filippakopoulos P, Qi J, Picaud S, Shen Y, Smith W, Fedorov O (2010). Selective inhibition of BET bromodomains. Nature.

[248] Amorim S, Stathis A, Gleeson M, Iyengar S, Magarotto V, Leleu X (2016). Bromodomain inhibitor OTX015 in patients with lymphoma or multiple myeloma: a dose-escalation, open-label, pharmacokinetic, phase 1 study. Lancet Haematol.

[249] Nilson K, Guo J, Turek M, Brogie J, Delaney E, Luse D (2015). THZ1 Reveals Roles for Cdk7 in Co-transcriptional Capping and Pausing. Mol Cell.

[250] Hu S, Marineau J, Rajagopal N, Hamman K, Choi Y, Schmidt D (2019). Discovery and Characterization of SY-1365, a Selective, Covalent Inhibitor of CDK7. Can Res.

[251] Chen H, Zheng J, Yan L, Zhou X, Jiang P, Yan F (2021). Super-enhancer-associated long noncoding RNA RP11–569A11.1 inhibited cell progression and metastasis by regulating IFIT2 in colorectal cancer. J Clin Lab Analysis.

[252] Natoni A, Murillo L, Kliszczak A, Catherwood M, Montagnoli A, Samali A (2011). Mechanisms of action of a dual Cdc7/Cdk9 kinase inhibitor against quiescent and proliferating CLL cells. Mol Cancer Ther.

